# Meso-Py: Dual Brain Cortical Calcium Imaging in Mice during Head-Fixed Social Stimulus Presentation

**DOI:** 10.1523/ENEURO.0096-23.2023

**Published:** 2023-12-15

**Authors:** Nicholas J. Michelson, Federico Bolaños, Luis A. Bolaños, Matilde Balbi, Jeffrey M. LeDue, Timothy H. Murphy

**Affiliations:** 1 Department of Psychiatry, Kinsmen Laboratory of Neurological Research, University of British Columbia, Vancouver, British Columbia V6T 1Z3, Canada; 2 Djavad Mowafaghian Centre for Brain Health, University of British Columbia, Vancouver, British Columbia V6T 1Z3, Canada

**Keywords:** cortex, GCaMP, mesoscale, mouse, social interaction, whisker

## Abstract

We present a cost-effective, compact foot-print, and open-source Raspberry Pi-based widefield imaging system. The compact nature allows the system to be used for close-proximity dual-brain cortical mesoscale functional-imaging to simultaneously observe activity in two head-fixed animals in a staged social touch-like interaction. We provide all schematics, code, and protocols for a rail system where head-fixed mice are brought together to a distance where the macrovibrissae of each mouse make contact. Cortical neuronal functional signals (GCaMP6s; genetically encoded Ca^2+^ sensor) were recorded from both mice simultaneously before, during, and after the social contact period. When the mice were together, we observed bouts of mutual whisking and cross-mouse correlated cortical activity across the cortex. Correlations were not observed in trial-shuffled mouse pairs, suggesting that correlated activity was specific to individual interactions. Whisking-related cortical signals were observed during the period where mice were together (closest contact). The effects of social stimulus presentation extend outside of regions associated with mutual touch and have global synchronizing effects on cortical activity.

## Significance Statement

We developed a system to provide a staged encounter between two head-fixed mice, allowing for simultaneous imaging of behavior and widefield dorsal cortical calcium activity in both animals throughout the encounter. In our experiments, neural signals between animals became more highly correlated during interaction periods, where physical touch between whiskers was possible. We also provide instructions and resources for investigators to develop standalone Raspberry Pi-based, mesoscale cortical calcium imaging systems. We anticipate that this method will provide a reproducible system to probe relationships between brain-to-brain activity during social interactions between mice.

## Introduction

Social interaction is a fundamental component of life across the animal kingdom. In mice, neurophysiological responses to social stimuli have been observed in many areas throughout the brain (Dölen et al., 2013; Gunaydin et al., 2014; Zhou et al., 2017; Rogers-Carter et al., 2018; Walsh et al., 2018; B. Guo et al., 2019; Sych et al., 2019; Tschida et al., 2019), but dorsal cortical circuits are relatively unexplored in this context. Widefield cortical calcium imaging samples neural activity across the entire dorsal cortex *in vivo* (Musall et al., 2019; Pinto et al., 2019; Gilad and Helmchen, 2020; Lohani et al., 2022; Matteucci et al., 2022), and may therefore present an opportunity to study mesoscale cortical circuit function during social behavior. However, finding neurophysiological correlates of interanimal interactions can be challenging because of the high degree of variability with which such interactions may occur. In this work, we employ a paradigm where cortical functional GCaMP (genetically encoded Ca^2+^ sensor) activity is imaged during staged, head-fixed interactions between mice. The head-fixed nature of the interaction allows for imaging of cortical activity across both interacting animals throughout the experiment, while reducing the complexity of possible interactions. Face to face interactions between mice synchronize cortical activity over spatial scales extending beyond single cortical areas and is not limited to regions primarily processing whisker/touch dependent signals. Moreover, we present detailed resources to help investigators set up a cost-effective, compact foot-print, and open-source Raspberry Pi-based imaging system that facilitates mesoscale head-fixed imaging in multiple interacting animals.

## Materials and Methods

### Animals and experimental considerations

All animal procedures were performed in accordance with the Canadian Council on Animal Care and approved by the University of British Columbia animal care committee’s regulations. Transgenic GCaMP6s tetO-GCaMP6s x CAMK tTA (Wekselblatt et al., 2016) were obtained from The Jackson Laboratory. All mice used in this study were males >60 d of age and housed in social housing (*n* = 15 mice up to four mice/cage from 6 cages) with 12/12 h light/dark cycles and free access to food and water. One pair of Thy1-GFP mice (Feng et al., 2000) from the same cage were also tested as controls to determine whether hemodynamic changes confounded the epifluorescence signal. We did not employ female mice or male and female mouse pairs because of potential for variation across the estrous cycle that may alter social behavior. Based on previous work we do expect female mice to show barrel cortex-dependent social interaction (Bobrov et al., 2014).

### Surgical procedure

**Figure 1.**
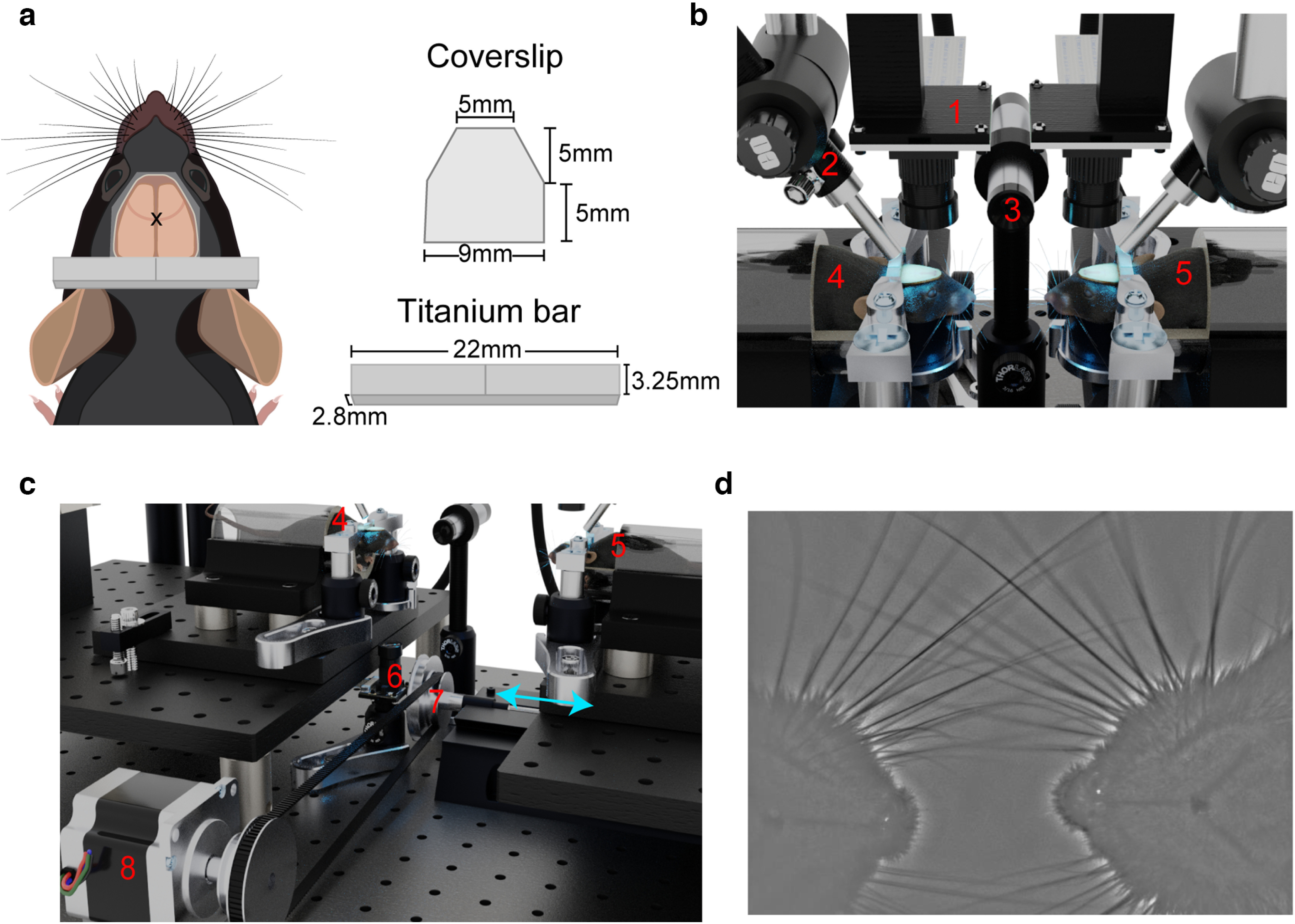
Setup for dual mouse brain imaging system. ***a***, Cartoon depiction of surgical preparation for transcranial mesoscale imaging, with custom cut coverslip and titanium bar for head fixation. x denotes the location of bregma. ***b***, Close-up view of mouse positioning during the interaction phase of the experiment. ***c***, Larger field of view render of the imaging system. Numbered components are as follows: (1) Raspberry Pi brain imaging camera; (2) GCaMP excitation and hemodynamic reflectance LED light guide; (3) ultrasonic microphone; (4) stationary mouse; (5) moving mouse; (6) Raspberry Pi infrared behavior camera; (7) stage translation knob; 8) stepper motor with belt controlling stage translation. Blue arrows indicate direction of motion of the translatable rail. ***d***, Example image of whisker overlap during a whisking event captured using a high-speed camera. Image is spatially high-pass filtered to accentuate whiskers.

Chronic windows were implanted on male mice that were at least eight weeks old, as previously described (Silasi et al., 2016). Mice were anesthetized with 2% isoflurane in oxygen, and fur and skin were removed from the dorsal area of the head, exposing the skull over the entire two dorsal brain hemispheres. After cleaning the skull with a phosphate buffered saline solution, a titanium head-fixing bar was glued to the skull above λ (Fig. 1*a*) and reinforced with clear dental cement (Metabond). A custom cut coverslip was glued with dental cement on top of the skull (Fig. 1*a*), with the edges of the window reinforced with a thicker mix of dental cement similar to the procedure of (Silasi et al., 2016). Mice recovered for at least 7 d before imaging or head-fixation.

### Social hierarchy measurements

Social rank was estimated using the tube-test assay (Fan et al., 2019) in a subset of eight mice from two cages. Briefly, mice were introduced to either end of a narrow Plexiglas tube (32 cm long, 2.5-cm inner diameter). Upon meeting in the middle, mice compete by pushing each other to get to the opposite side. The mouse which pushes the other back out of the tube is deemed the winner. A plastic barrier was inserted at the midpoint of the tube and was removed when both mice approached the middle to avoid biases. All combinations of mice within a cage were tested in a round robin format to determine the linear hierarchy. Tube test tournaments were repeated for four trials.

### Dual mouse imaging experiments

Two Raspberry Pi imaging rigs were set up facing each other, and initially separated by 14 cm. A parts list and assembly instructions for the Raspberry Pi widefield imaging rig are included in the Open Science Framework project (see link below, in Resource availability section). One imaging rig was placed atop a translatable rail (Sherline 5411 XY Milling Machine Base), which was driven by a stepper motor to bring the mouse (hereafter referred to as the moving mouse) into the proximity of the other mouse (stationary mouse, 6- to 12-mm intersnout distance; see Table 1 and Open Science Framework project for details). Stage translation occurred 2 min after imaging began, and lasted ∼27.5 s. Animals were imaged together for 2 min before they were returned to their separated orientation, and imaged for another 2 min. Thus, we imaged dorsal cortical activity from two head-restrained mice simultaneously, while varying the distance between snouts (Fig. 1*b*,*c*; Movie 1). Randomization was not used to allocate animals into groups because of the relatively small sample size of mice, however, all combinations of cagemate mouse pairs were tested. Cagemate and noncagemate designations were determined early at weaning before any experiments. In some experiments, a partition, either a copper mesh or an opaque cardboard sheet, was placed in front of the stationary mouse and remained there throughout the experiment. Each partition prevents physical contact between whiskers; however, the mice can likely see each other through the copper mesh, and smell each other through either partition. Mice were habituated to the system for at least one week before conducting experiments by head-fixing the animals each day and performing all procedures [e.g., translation, switching light-emitting diodes (LEDs) on/off] without the presence of the other mouse.

**Table 1:** Parts list for social interaction system

Description	#	Manufacturer	Part number
Aluminum Breadboard 18 × 24 × 1/2”, 1/4”−20 Taps	1	Thorlabs	MB1824
Ø1” Pillar Posts with 1/4”−20 Taps, 2”	4	Thorlabs	RS2
Ø1” Pillar Posts with 1/4”−20 Taps, 3”	8	Thorlabs	RS3
Ø1” Pillar Posts with 1/4”−20 Taps, 6”	8	Thorlabs	RS6
Clamping Fork, 1.24” Counterbored Slot, Universal	4	Thorlabs	CF125
Ø1/2” Pedestal Post Holder	3	Thorlabs	PH2E
Ø1/2” Optical Post, SS, 8–32 Setscrew, 1/4”−20 Tap, L = 8”	3	Thorlabs	TR8
Ø1/2” Optical Post, SS, 8-32 Setscrew, 1/4”−20 Tap, L = 12”	1	Thorlabs	TR12
Right-Angle Clamp for Ø1/2” Posts, 3/16” Hex	2	Thorlabs	RA90
Ø25 mm Post Spacer, Thickness = 3 mm	1	Thorlabs	RS3M
RPi Camera (F), Supports Night Vision, Adjustable-Focus	2	Waveshare	10299
Flex Cable for Raspberry Pi Camera or Display - 2 m	2	Adafruit	2144
Triple light guide and imaging parts			
Triple Bandpass Filter (camera)	1	Chroma	69013m
Liquid Light Guide	1	Thorlabs	LLG0338-4
SM1 Adapter for Liquid Light Guide	1	Thorlabs	AD3LLG
SM1 Lens Tube, 3.00” Thread Depth	3	Thorlabs	SM1L30
SM1 Lens Tube, 1.00” Thread Depth	3	Thorlabs	SM1L10
SM1 Lens Tube, 2.00” Thread Depth	1	Thorlabs	SM1L20
SM1 Retaining Rings	2	Thorlabs	SM1RR-P10
Dichroic Cage Cube	2	Thorlabs	CM1-DC
Cage Cube Connector	1	Thorlabs	CM1-CC
Compact Clamp with Variable Height	1	Thorlabs	CL3
Bi-Convex Lens	4	Thorlabs	LB1761
AT455DC size: 26 × 38 mm	1	Chroma	AT455DC
25 × 36 mm Longpass Dichroic Mirror, 550 nm Cutoff	1	Thorlabs	DMLP550R
Ø1” Bandpass Filter, CWL = 620 ± 2 nm, FWHM = 10 ± 2 nm	1	Thorlabs	FB620-10
ET480/30× size: 25 mmR R=Mounted in Ring	1	Chroma	ET480/30x
Ø1” Bandpass Filter, CWL = 440 ± 2 nm, FWHM = 10 ± 2 nm	1	Thorlabs	FB440-10
Royal-Blue (448 nm) Rebel LED	1	Luxeon Star	SP-01-V4
Blue (470 nm) Rebel LED	1	Luxeon Star	SP-01-B6
Red-Orange (617 nm) Rebel LED	1	Luxeon Star	SP-01-E6
Machined parts (stainless steel)			
Milled as-1.50_2_v2.SLDPRT	3		
Spacer_with_wire_hole_as-.500_v2.SLDPRT	3		
LED_mount_as-1.50_v2.SLDPRT	3		
3D-printed parts (black PLA)			
TripleLEDLightGuide_Base.stl	1		
Light_Guide_Mount_V2.stl	1		

10.1523/ENEURO.0096-23.2023.video.1 Movie 1. Cortical signal responses for interacting mice. Data from two head-restrained tTA-GCaMP6s mice at the onset of the interaction phase. The two pseudo-colored images show the processed calcium images from the left and right mouse. The color bar indicates the ΔF/F_0_ of the signals. Behavior video is shown below.

The entire imaging system was housed inside a box lined with acoustic foam which reduced ambient light and noise. Throughout the experiment, audio recordings were obtained at 200 kHz using an ultrasonic microphone (Dodotronic, Ultramic UM200K) positioned within the recording chamber ∼5 cm from each mouse’s snout. Audio recordings were analyzed for ultrasonic vocalizations using the MATLAB toolbox DeepSqueak (Coffey et al., 2019).

### Behavior imaging

**Figure 2.**
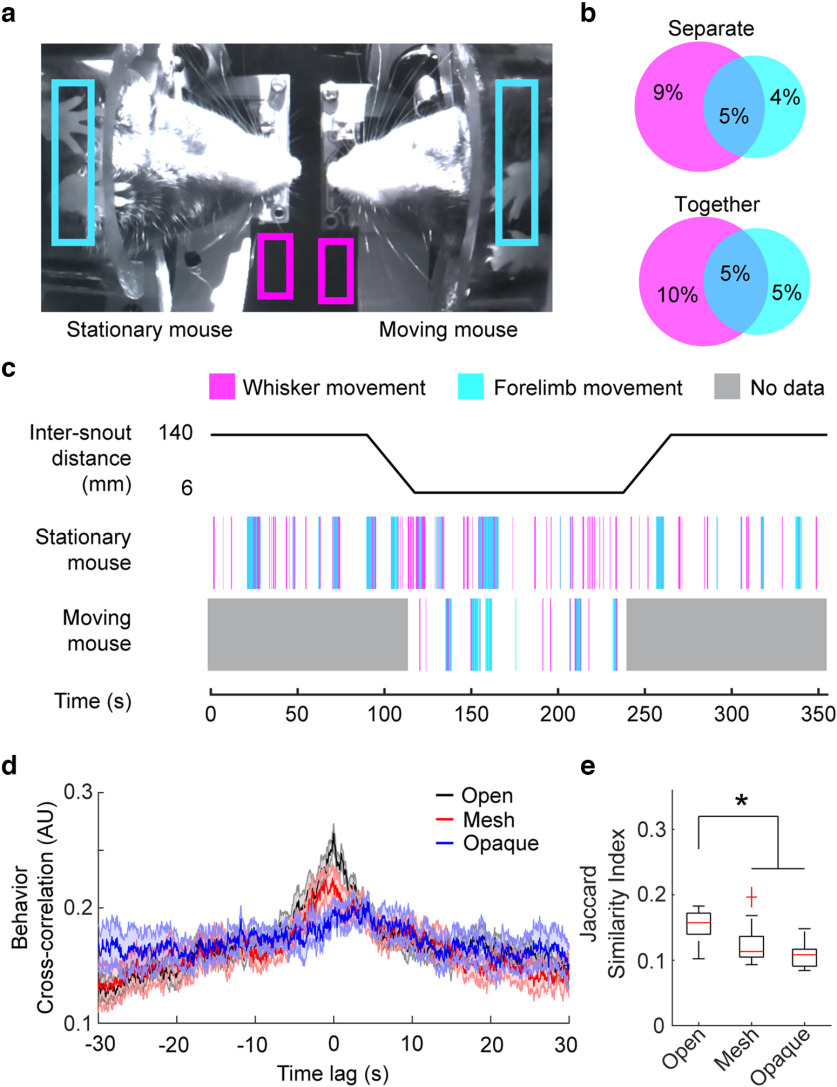
Mice coordinate behavior during interaction. ***a***, Example infrared (IR) bottom camera image of both mice during the interaction phase of the experiment. Regions over each mouse’s whiskers and forelimbs are shown in magenta and cyan boxes, respectively, to estimate motion. ***b***, Average percentage of time spent behaving during the first separated phase of the experiment (top) and the interaction phase (bottom). Intersecting regions show concurrent whisker and forelimb movements (∼5% of total time). ***c***, Timeline of experimental paradigm (top) and ethograms for the stationary and moving mouse (bottom). ***d***, Cross-correlation of each mouse’s binary behavior vectors during the interaction phase where whiskers could freely touch open (black) versus during trials where a mesh (red) or opaque (blue) barrier were placed between the animals, preventing physical touch. Behaviors across mice during the interaction phase were significantly correlated near 0 lag when there was no barrier present (open condition). ***e***, Intersection over union (Jaccard similarity index) for the behavior vectors was significantly greater during the interaction phase across mice for trials with no barriers present compared with mesh or opaque barrier trials. *n* = 33 mouse pairs; one-way ANOVA with *post hoc* Bonferroni test for multiple comparisons; *F*_(2,42)_ = 10.1; *p* = 2.6 × 10^−4^. See Extended Data Figures 2-1 and 2-2 for additional data. Asterisk indicates significance.

The experimental setup was illuminated with an infrared (850 nm) light-emitting diode (LED), and behaviors were monitored using an infrared Raspberry Pi camera (OmniVision, OV5647 CMOS sensor). Behavior videos were captured at a framerate of 90 frames per second (fps) with a resolution of 320 × 180 pixels. The camera was positioned such that the stationary mouse was always included in the field of view and both mice were visible when they were together (Fig. 2*a*).

10.1523/ENEURO.0096-23.2023.f2-1 Extended Data Figure 2-1 Protocol for estimating behaviors. ***a***, Raw motion energy within the whisker region of interest. ***b***, Smoothed whisker motion energy. Motion energy exceeding a threshold of the mean + 1 SD (red line) is classified as binary movement behavior (shaded areas). ***c***, Example montage of whisking behavior. Images are taken from beneath the mouse (see Figs. 1 and 2). Individual frames are cropped and displayed with saturated pixels, and a Canny edge detection algorithm was run over the whisker region to enhance visualization of whiskers. A whisker protraction event can be seen at 0.14 s. ***d–f***, Same as ***a–c*** for forelimb movements. Individual frames are cropped to aid with visualization. Left and right paws are labelled with red and cyan markers, respectively. Download Figure 2-1, TIF file.

10.1523/ENEURO.0096-23.2023.f2-2 Extended Data Figure 2-2 Number of whisker or forelimb movements do not change between trial phases. Number of whisker movements (left) and forelimb movements (right) for all trial phases during open social interaction experiments (***a***) and barrier controls (***b***, ***c***). During: during interaction period while mice are stationary and together (face to face); before/after: before/after interaction period while mice are stationary and apart. No significance between trial phases for all conditions (open *n* = 33 trials, mesh *n* = 16 trials, opaque *n* = 11 trials). Download Figure 2-2, TIF file.

### GCaMP image acquisition

GCaMP activity was imaged using RGB Raspberry Pi Cameras (OmniVision OV5647 CMOS sensor). The GCaMP imaging cameras had lenses with a focal length of 3.6 mm with a field of view of ∼10.2 × 10.2 mm, leading to a pixel size of ∼40 μm, and were equipped with triple-bandpass filters (Chroma 69013m), which allowed for the separation of GCaMP epifluorescence signals and reflectance signals into the green and blue channels, respectively. Twenty-four-bit RGB images of GCaMP activity and reflectance were captured at 28.9 fps and 256 × 256 resolution. The three cameras (two brain and one behavior) were configured such that one camera was used to start the acquisition of the other two.

While mesoscale GCaMP imaging can be achieved with single wavelength illumination (Gilad and Helmchen, 2020; Nakai et al., 2023; Ramandi et al., 2023), in this experiment, each cortex was illuminated using two LEDs simultaneously, where one light source (short blue, 447.5 nm Royal Blue Luxeon Rebel LED SP-01-V4 with Thorlabs FB 440-10 nm band pass filter) provides information about light reflectance during hemodynamic changes (Xiao et al., 2017), and the other light source (long blue, 470 nm Luxeon Rebel LED SP-01-B6 with Chroma 480/30 nm) excites GCaMP for green epifluorescence. For each mouse, the light from the excitation and hemodynamic reflectance LEDs were directed into a single liquid light guide which was positioned to illuminate the cortex (Fig. 1*b*). Further details can be found in the Parts List and assembly instructions document found in the Open Science Repository (https://osf.io/96bqv). Each LED was driven by a custom LED driver which was triggered by the Raspberry Pi to turn on at the start of the trial and off at the end of the trial. This sudden change in illumination was used during *post hoc* analysis to synchronize frames across cameras. With the current recording setup, the Raspberry Pi cameras occasionally drop frames (on average 0.1% of frames) as a result of writing the data to the disk. We identified the location of dropped frames by tagging each frame with a timestamp and found that consecutive frames were rarely dropped. Given the small number of dropped frames, and the relatively slow kinetics of GCaMP6s (Chen et al., 2013), the lost data were estimated by interpolating the signal for each pixel.

### GCaMP image processing

Image preprocessing was conducted with Python using a Jupyter Notebook (Kluyver et al., 2016). Further analysis was conducted using MATLAB (The MathWorks). Green and blue channels, which contain the GCaMP6s fluorescence and the blood volume reflectance signals, respectively (Ma et al., 2016; Wekselblatt et al., 2016; Valley et al., 2020), were converted to ΔF/F_0_. The baseline image, estimated as the mean image across time for the entire recording, was subtracted from each individual frame (ΔF). The result of this difference was then divided by the mean image, yielding the fractional change in intensity for each pixel as a function of time (ΔF/F_0_).

To correct for potential hemodynamic artifacts (Ma et al., 2016), blue light (440 ± 5 nm) reflectance ΔF/F_0_ was subtracted from the green fluorescence ΔF/F_0_. In this way, small changes in the brain reflectance because of blood volume changes do not influence the epifluorescence signal. While we acknowledge that a green reflectance strobing and model-based correction may be advantageous (Ma et al., 2016), certain technical aspects of the Raspberry Pi camera such as its rolling shutter and inability to read its frame exposure clock prevent this method from being implemented. The short blue wavelength (447 nm) with 440 ± 5-nm filter is close to an oxy/deoxygenated hemoglobin isosbestic point, and the reflected 447-nm light signal was found in prior work to correlate well with reflected 530-nm green light signal (Xiao et al., 2017), which is typically used to estimate signal changes resulting from hemodynamic activity (Ma et al., 2016). Moreover, the 447-nm LED produces very little green epifluorescence signal at the power used to assess reflectance from the dorsal cortical surface, and expected hemodynamic changes are relatively small compared with the signal-noise-ratio of GCaMP6s (Dana et al., 2014). Examples of corrected and uncorrected signals using this method can be seen in prior work (Xiao et al., 2017; Murphy et al., 2020).

Occasionally, noisy extreme pixel values for ΔF/F_0_ were observed because of imaging near the edge of the window or because of small F0 in the denominator of ΔF/F_0_. To reduce their contribution, pixels exceeding a threshold value were set to be equal to the threshold, thereby reducing artifacts from smoothing or filtering that might result from inclusion of abnormally large ΔF/F_0_ values. The threshold was set at the mean ± 3.5× the SD of each pixel’s time-series for GCaMP data, and at 15% ΔF/F_0_ for the reflectance data (which is much larger than expected reflectance signal values). The ΔF/F_0_ signal was then smoothed with a Gaussian image filter (σ = 1) and filtered using a fourth order Butterworth bandpass filter (0.01–12.0 Hz; Vanni and Murphy, 2014).

### Behavior quantification

To extract behavior events, a region of interest (ROI) was manually drawn on the behavior video over each mouse’s whiskers and forelimbs (Fig. 2*a*). The motion energy within each ROI was calculated by taking the absolute value of the temporal gradient of the mean pixel value within the ROI. The resulting motion energy was smoothed via convolution with a Gaussian kernel (σ = 25 frames) and a threshold was established at the mean + 1 SD to detect behaviors (Extended Data Fig. 2-1*a*). This analysis captured whisker and forelimb movements for the stationary mouse for the entirety of the experiment, and for the moving mouse only during the interaction phase (Fig. 2*c*; Extended Data Fig. 2-1*b*), when the moving mouse was in the behavior frame. Behavior data from two trials were excluded because of poor illumination, resulting in the inability to resolve whisker movements.

### Interbrain correlation analysis

**Figure 3.**
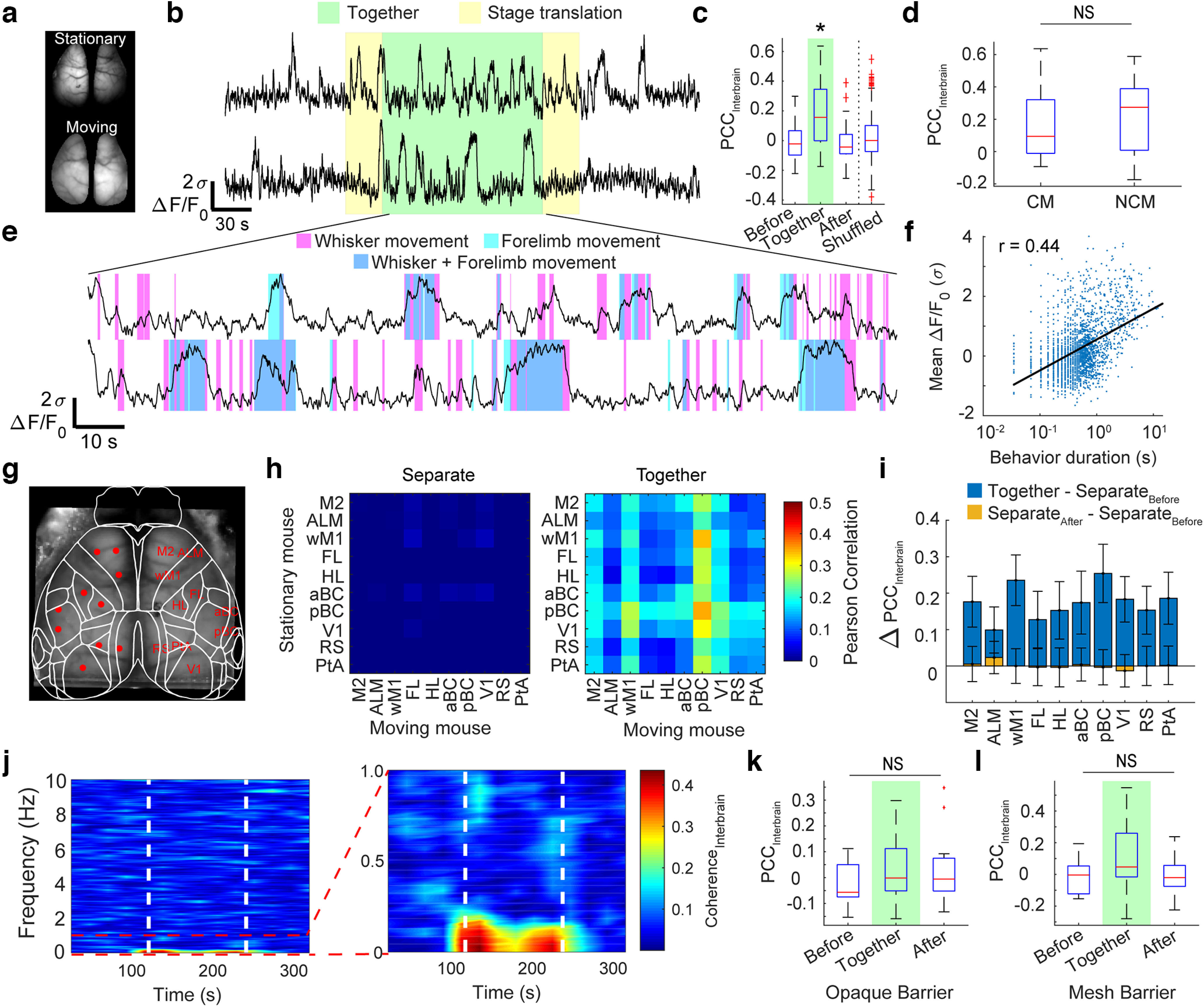
Interbrain synchronization during interaction. ***a***, Example images of dorsal cortical windows for stationary mouse (top) and moving mouse (bottom). ***b***, Representative example of median GCaMP6s activity across the cortical mask for each mouse. ***c***, Pearson correlation coefficients of the two global signals were significantly greater during the interaction phase than either of the two separate phases (*n* = 35 mouse pairs, *p* < 0.001; repeated measures ANOVA with *post hoc* Bonferroni correction for multiple comparisons). Interbrain correlations during interaction were significantly greater than trial-shuffled interaction-phase pairings (*n* = 35 mouse pairs vs *n* = 595 shuffled mouse pairs, *p* < 0.001; *t* test). ***d***, Interbrain signal correlation does not depend on cagemate (CM) versus noncagemate (NCM) experiments (*t* test, *p* = 0.66, cagemates *n* = 20 mouse pairs, noncagemates *n* = 13 mouse pairs). ***e***, Expanded view of global signals during interaction phase with behavior annotations overlaid. ***f***, Global cortical signal ΔF/F_0_ is positively correlated with duration of whisking or forelimb movement (Spearman correlation coefficient; *r* = 0.40; *p* < 0.001). ***g***, Example image of transcranial mask with putative cortical regions labeled. Abbreviations: ALM, anterior lateral motor cortex; M2, secondary motor cortex; wM1, whisker motor cortex; aBC, anterior barrel cortex; pBC, posterior barrel cortex; HL, hindlimb; FL; forelimb; lPTA; lateral parietal association area; RS, retrosplenial cortex; V1, primary visual cortex. ***h***, Averaged interbrain correlation matrices across all experiments during the period before interaction (left) and the period during interaction (right). ***i***, Change in interbrain correlation for each region of interest against all other regions, averaged across mice (*n* = 35 mouse pairs, *p < 0.05; two-way ANOVA with *post hoc* Tukey–Kramer test). Bars show mean ± SE. ***j***, Time-varying interbrain coherence, computed with a 45-s window on the global cortical signals, and averaged across all experiments, shows an increase in coherence from 0 to 0.2 Hz during the interaction phase (white dashed lines). ***k***, ***l***, Pearson correlation coefficients of the two global signals showed no significant difference between trial phases when an opaque partition (***k***; *p* = 0.34, repeated measures ANOVA) or copper mesh (***l***; *p* = 0.88, repeated measures ANOVA) were placed between the mice, preventing whisker contact. Asterisks indicate significance. NS indicates not significant. See Extended Data Figures 3-1, 3-2, 3-3, and 3-4 for additional data.

Correlation across brains was calculated using the Pearson’s correlation coefficient (PCC). To compare correlations across trial phases, the interbrain PCC was calculated for a 1-min period during initial-separate, together, and final-separate trial phases. Global signals were calculated as the median ΔF/F_0_ across the entire dorsal cortex. Time-varying coherence between global signals was estimated with multitaper methods over a 45-s window with 22.5-s overlap using the MATLAB Chronux toolbox with a time-bandwidth product of 5 and a taper number of 9 (Bokil et al., 2010; Mitra and Bokil, 2009). Individual regions were selected from coordinates with respect to bregma (Fig. 3*g*), and their placement was verified after alignment with the Allen Institute Common Coordinate Framework brain atlas (Wang et al., 2020; Saxena et al., 2020), which used key-points along bregma, the superior sagittal sinus, and the space between the olfactory bulb and the frontal cortex. It should be noted that while these key-points can provide a reasonable alignment of the data with the cortical atlas, using key-points obtained with functional mapping may be a more accurate method. These coordinates yielded expected functional networks (Vanni et al., 2017) as identified by calculating seed pixel correlation maps (Extended Data Fig. 3-2). Region-specific time-series data were calculated as the median activity within a five by five-pixel area within each region location.

10.1523/ENEURO.0096-23.2023.f3-1 Extended Data Figure 3-1 No interanimal correlation observed in Thy1-GFP mice. ***a***, Representative example of GFP activity global signals over the entire cortical mask for the stationary mouse (top) and moving mouse (bottom). Green shading indicates period when mice were together. ***b***, Pearson correlation coefficients computed at each phase of the experiment. Dashed line shows median correlation coefficient between global signals for the GCaMP mice during the interaction phase of the experiment from Figure 3*c*. No significant difference was observed between phases. *n* = 4 trials, *p* = 0.4, one-way ANOVA. Download Figure 3-1, TIF file.

10.1523/ENEURO.0096-23.2023.f3-2 Extended Data Figure 3-2 Seed pixel correlation maps for putative cortical ROIs from an example mouse. Pearson correlation coefficients over the entire trial (spanning both separate and together phases) were calculated between every pixel within the cortical mask and the averaged signal obtained from a five by five-pixel neighborhood chosen from the specified region in each panel. Scale bar: 2 mm. Download Figure 3-2, TIF file.

10.1523/ENEURO.0096-23.2023.f3-3 Extended Data Figure 3-3 Intrabrain correlations increase during interaction phase of the trial. ***a***, Averaged intrabrain correlation matrices across all experiments during the period before interaction (left) and the period during interaction (right). ***b***, Change in intrabrain correlation for each region of interest against all other regions, averaged across mice (*n* = 35 mouse pairs, *p < 0.05; two-way ANOVA with *post hoc* Tukey–Kramer test). Bars show mean ± SE; *y*-axis is scaled similarly to interbrain correlation changes from Figure 3*i*. Download Figure 3-3, TIF file.

**Figure 4.**
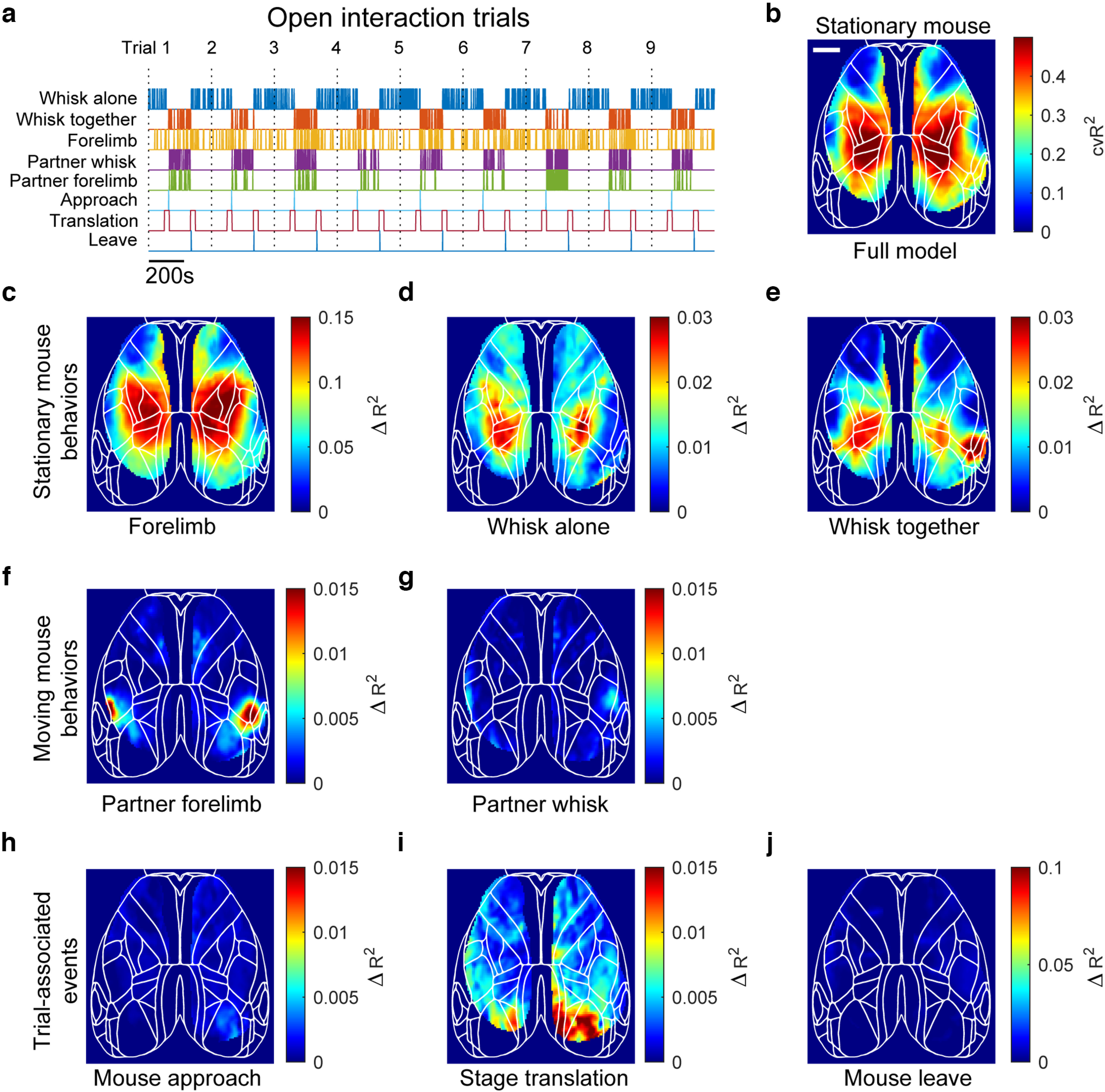
Ridge regression model in open interaction trials confirms somatotopic representation of whisker and forelimb behaviors across animals. ***a***, Binary event vectors (colored lines) considered in the ridge regression model. Model included nine separate interaction trials which had been concatenated together (dotted lines; partner limb and whisker behavioral variables were only assessed during the together phase). ***b***, Explained variance for the full model after 10-fold cross-validation, projected back onto the cortical map. Scale bar: 2 mm. ***c–e***, Unique contribution for each stationary mouse behavioral model variable; taken as the difference in explained variance between the full model and the reduced model with the specified variable randomly permuted. ***f***, ***g***, Same as ***c–e***, except for the partner mouse behaviors. ***h–j***, Same as ***c–e***, except for trial-associated events. See Extended Data Figures 4-1, 4-2, 4-3, and 4-4 for additional data.

10.1523/ENEURO.0096-23.2023.f3-4 Extended Data Figure 3-4 No ultrasonic vocalizations detected during social-interaction tests. Example data from the social interaction experiment (top), compared to a control experiment taken from a breeder mouse introduced to a female (bottom). Ultrasonic vocalizations are clearly observed in the female stimulus control experiment, but not in the two-mouse imaging experiments. Download Figure 3-4, TIF file.

### Ridge regression analysis

Analysis followed the protocol and incorporated MATLAB code provided previously (Musall et al., 2019). ΔF/F_0_ data from the stationary mouse from four consecutive trials were brain masked, concatenated, and then reduced to the first 200 principal components using singular value decomposition (Musall et al., 2019). Timestamps of relevant behavior events from the stationary mouse (whisker movement alone, whisker movement together, forelimb movement) and the moving mouse (whisker movement together, forelimb movement together), and trial-associated events (stage translation on, moving mouse approach, moving mouse leave) corresponding to each trial were also concatenated (Fig. 4*a*). A design matrix (Musall et al., 2019) was then created for each of these binary event variables, where behavior events included every sample after the event up to 2 s, mouse approach and mouse leaving events included 5 s before or after the approach or leave event, respectively, and the stage translation events included every sample after the event up to 2 s. The design matrix was then fitted against the reduced neural data using ridge regression and the variance explained was obtained with 10-fold cross-validation. To estimate the explained variance of each model variable individually, a reduced model was created where the specified model variable was randomly permuted (Musall et al., 2019), and the explained variance was computed for this reduced model. The difference in explained variance between the full model and the reduced model shows the unique contribution of the specified model variable.

10.1523/ENEURO.0096-23.2023.f4-1 Extended Data Figure 4-1 Ridge regression model on an additional mouse in open interaction trials. ***a–j***, Binary event vectors (colored lines) considered in the ridge regression model. Model included three separate interaction trials which had been concatenated together (dotted lines; partner limb and whisker behavioral variables were only assessed during the together phase). ***b***, Explained variance for the full model after 10-fold cross-validation, projected back onto the cortical map. Scale bar: 2 mm. ***c–e***, Unique contribution for each stationary mouse behavioral model variable; taken as the difference in explained variance between the full model and the reduced model with the specified variable randomly permuted. ***f***, ***g***, Same as ***c–e***, except for the partner mouse behaviors. ***h–j***, Same as ***c–e***, except for trial-associated events. Download Figure 4-1, TIF file.

10.1523/ENEURO.0096-23.2023.f4-2 Extended Data Figure 4-2 Ridge regression model on an additional mouse in open interaction trials. ***a–j***, Binary event vectors (colored lines) considered in the ridge regression model. Model included two separate interaction trials which had been concatenated together (dotted lines; partner limb and whisker behavioral variables were only assessed during the together phase). ***b***, Explained variance for the full model after 10-fold cross-validation, projected back onto the cortical map. Scale bar: 2 mm. ***c–e***, Unique contribution for each stationary mouse behavioral model variable; taken as the difference in explained variance between the full model and the reduced model with the specified variable randomly permuted. ***f***, ***g***, Same as ***c–e***, except for the partner mouse behaviors. ***h–j***, Same as ***c–e***, except for trial-associated events. Download Figure 4-2, TIF file.

10.1523/ENEURO.0096-23.2023.f4-3 Extended Data Figure 4-3 Ridge regression model in mesh barrier trials. ***a***, Binary event vectors (colored lines) considered in the ridge regression model. Model included four separate interaction trials which had been concatenated together (dotted lines; partner limb and whisker behavioral variables were only assessed during the together phase). ***b***, Explained variance for the full model after 10-fold cross-validation, projected back onto the cortical map. Scale bar: 2 mm. ***c–e***, Unique contribution for each stationary mouse behavioral model variable; taken as the difference in explained variance between the full model and the reduced model with the specified variable randomly permuted. ***f***, ***g***, Same as ***c–e***, except for the partner mouse behaviors. ***h–j***, Same as ***c–e***, except for trial-associated events. Download Figure 4-3, TIF file.

10.1523/ENEURO.0096-23.2023.f4-4 Extended Data Figure 4-4 Ridge regression model on an additional mouse in mesh barrier trials. ***a–j***, Binary event vectors (colored lines) considered in the ridge regression model. Model included four separate interaction trials which had been concatenated together (dotted lines; partner limb and whisker behavioral variables were only assessed during the together phase). ***b***, Explained variance for the full model after 10-fold cross-validation, projected back onto the cortical map. Scale bar: 2 mm. ***c–e***, Unique contribution for each stationary mouse behavioral model variable; taken as the difference in explained variance between the full model and the reduced model with the specified variable randomly permuted. ***f***, ***g***, Same as ***c–e***, except for the partner mouse behaviors. ***h–j***, Same as ***c–e***, except for trial-associated events. Download Figure 4-4, TIF file.

### Statistics

Statistical tests (Table 2) were conducted with MATLAB. All data were tested for normality using a Kolmogorov–Smirnov test before subsequent statistical analyses. Correlation values were transformed using Fisher’s z-transformation. Comparisons between two groups were conducted using two-tailed *t* tests for parametric data and Wilcoxon signed-rank tests for nonparametric data. Comparisons between trial phases were assessed using a repeated measures ANOVA with *post hoc* Bonferroni correction for multiple comparisons. Results are presented as mean ± SD. All statistically significant results were observed on the GCaMP signals alone as well as the hemodynamic corrected signals.

**Table 2:** Statistical table of all analyses

	Data structure	Type of test	Test values and power
Figure 2*d*	All groups normally distributed	One-way ANOVA with *post hoc* Bonferroni multiple comparisons test	*F*_(2,42)_ = 10.2; *p* = 2.4 × 10^–4^
Figure 2*e*	All groups normally distributed	One-way ANOVA with *post hoc* Bonferroni multiple comparisons test	*F*_(2,42)_ = 10.1; *p* = 2.6 × 10^–4^
Figure 3*c*	All groups normally distributed	Repeated measures ANOVA with Bonferroni’s multiple comparisons test	*F*_(2,68)_ = 11.2; *p* = 0.002
Figure 3*d*	All groups normally distributed	Two sample *t* test	*t*_(33)_ = −0.44; *p* = 0.66
Figure 3*f*	Groups not normally distributed	Spearman correlation coefficient	*r* = 0.40; *p* = 6.0 × 10^−81^
Figure 3*i*	All groups normally distributed	Two-way ANOVA with *post hoc* Bonferroni test	Trial phase: *F*_(1,680)_ = 136; *p* = 1.0 × 10^−28^
			Brain region: *F*_(9,680)_ = 0.81; *p* = 0.61
			Interaction: *F*_(9,680)_ = 1.2; *p* = 0.30
Figure 3*k*	All groups normally distributed	Repeated measures ANOVA	*F*_(2,28)_ = 1.0; *p* = 0.34
Figure 3*l*	All groups normally distributed	Repeated measures ANOVA	*F*_(2,30)_ = 0.22; *p* = 0.88
Extended Data Figure 2-2*a*	All groups normally distributed	One-way ANOVA	Whisker movements: *F*_(2,51)_ = 0.68; *p* = 0.51
			Forelimb movements: *F*_(2,51)_ = 1.02; *p* = 0.37
Extended Data Figure 2-2*b*	All groups normally distributed	One-way ANOVA	Whisker movements: *F*_(2,45)_ = 0.28; *p* = 0.76
			Forelimb movements: *F*_(2,45)_ = 0.41; *p* = 0.67
Extended Data Figure 2-2*c*	All groups normally distributed	One-way ANOVA	Whisker movements: *F*_(2,30)_ = 0.85; *p* = 0.44
			Forelimb movements: *F*_(2,30)_ = 0.05; *p* = 0.95
Extended Data Figure 3-1*b*	All groups normally distributed	One-way ANOVA	*F*_(2,9)_ = 0.91; *p* = 0.44
Extended Data Figure 3-3*b*	All groups normally distributed	Two-way ANOVA with *post hoc* Bonferroni test	Trial phase: *F*_(1,680)_ = 44.1, *p* = 6.3 × 10^–11^
			Brain region: *F*_(9,680)_ = 0.30, *p* = 0.98
			Interaction: *F*_(9,680)_ = 0.01, *p* = 1.0

### Resource availability

Resources to assist in building cortex-wide GCaMP imaging systems, including parts lists, assembly instructions, and CAD files are available at the Open Science Framework project entitled Dual Brain Imaging (https://osf.io/afuzn/). Code for image acquisition, preprocessing, and analysis are available at the University of British Columbia’s Dynamic Brain Circuits in Health and Disease research cluster’s GitHub (https://github.com/ubcbraincircuits/dual-mouse). Data are available in the Federated Research Data Repository at https://doi.org/10.20383/101.0303.

## Results

### Meso-Py

We provide a flexible open-source system to capture mouse mesoscale cortical optical signals. The system is built around a robust 3D positioning system employing inexpensive milling machine X, Y, and Z translators. We provide Python-based software and open-source hardware that makes extensive use of 3D-printed parts and off-the-shelf optics to provide cost-effective imaging systems sufficient to measure GCaMP signals. An advantage of the compact nature of the imaging systems is that they can be placed in close proximity to one another, allowing capture of optical signals from two mice that move in relation to each other. Having one imaging system mounted on a rail and the other stationary allows the creation of staged and constrained interactions that are well suited for examining interbrain correlated activity. Our experiments consisted of three major temporal periods: mice apart, together where nose-to-nose distance was 6–12 mm, and apart again. Most analysis was performed while mice were stationary and apart, or together and not moving. Based on published studies of whisker geometry, we estimate that the whiskers of the two mice would contact each other over 12 mm of nose to nose spacing (Carvell and Simons, 2017). We also confirmed this using high-speed video where individual whiskers could be measured and overlap confirmed (Fig. 1*d*).

### Mice exhibit correlated bouts of behavior

Forelimb and whisker movements were monitored for each mouse to measure behavior (Fig. 2*a*) using a camera positioned underneath the interacting mice (Fig. 1*c*). The stationary mouse’s behavior was captured throughout the entire experiment, while the moving mouse’s behavior acquisition was limited to the interaction period only (Fig. 2*c*). Bouts of forelimb and whisker movements often occur simultaneously (Fig. 2*b*), and the amount of time spent actively moving whiskers or forelimbs, expressed as percentage of time spent behaving in each trial phase, did not change between the separate period and the interaction period (*n* = 33 trials, 14.1 ± 3.4% whisking separate vs 14.4 ± 4.3% whisking interaction, and 8.9 ± 3.8% forelimb separate vs 9.8 ± 4.8% forelimb interaction period, *n* = 33 trials, *p* = 0.77, paired *t* test; Fig. 2*b*), and the percentage of time spent whisking was not found to be significantly different when compared with a subset of trials where the partner mouse was either absent or kept at the full distance for the entirety of the trial (*n* = 7 trials, 15.2 ± 3.0% whisking alone or separated vs *n* = 33 trials, 14.4 ± 4.3% whisking during interaction; *p* = 0.69, Wilcoxon rank-sum test). The total number of behavior events did not differ across trial phases as well (Extended Data Fig. 2-2). To assess the temporal relationship between behaviors across animals, the cross-correlation was computed. The cross-correlation can be interpreted as an estimate of the correlation between two signals as one signal is shifted in time, resulting in a measure of similarity between the two signals as a function of time shifts (e.g., time lag). Behaviors across mice exhibited temporal coordination as shown by the peak in the cross-correlation of binary behavior vectors at time lag 0 s that exhibited a rise and fall over 20 s (Fig. 2*d*, black). The cross-correlation of behavior vectors across mice with mesh (Fig. 2*d*, red) or opaque (Fig. 2*d*, blue) barriers placed between them was significantly reduced at lag 0 compared with trials without barriers where whisker touch is possible (one-way ANOVA with *post hoc* Bonferroni test, *F*_(2,42)_ = 10.2, *p* = 2 × 10^−4^, open *n* = 33 trials, mesh *n* = 16 trials, opaque *n* = 11 trials; Fig. 2*d*). Intersection over union for the two behavior vectors, which measures the ratio of shared behavior events (e.g., A and B behave) to all behavior events (e.g., A or B behave), was significantly reduced across animals when mesh or opaque barriers were placed between them (one-way ANOVA with *post hoc* Tukey–Kramer test, *F*_(2,42)_ = 10.1, *p* = 2.6 × 10^−4^, open *n* = 33 trials, mesh *n* = 16 trials, opaque *n* = 11 trials; Fig. 2*e*).

### Interbrain synchronization across multiple cortical regions

Global signals were calculated as the spatial median ΔF/F_0_ across the entire masked region of the two cortical hemispheres (Fig. 3*a*,*b*). The Pearson’s correlation coefficient (PCC) between global signals from each mouse was significantly higher during the interaction phase (0.19 ± 0.23 interaction phase) of the experiment than during either of the two separate phases (−0.007 ± 0.13 before; −0.01 ± 0.14 after; repeated measures ANOVA, *n* = 35 trials, trial-phase: *F*_(1,33)_ = 11.2, *p* = 0.002; Fig. 3*c*). Interbrain PCCs during the interaction phase were also significantly higher than PCCs calculated across trial-shuffled global signal pairings during the interaction phase (0.19 ± 0.23 interaction phase vs 0.02 ± 0.14 trial-shuffled, *n* = 35 correct pairs vs *n* = 595 shuffled pairs, *p* = 1.2 × 10^−11^, two sample *t* test; Fig. 3*c*). Cagemate versus noncagemate pairings did not have a significant effect on interbrain correlation (two-sample *t* test, *p* = 0.66; Fig. 3*d*). A subset of the experiments included back-to-back repeat pairings of the same animals. PCCs were not found to be significantly different between first and second pairings (0.22 ± 0.28, *n* = 6 first pairings; 0.19 ± 0.15, *n* = 6 s pairings; *p* = 0.82, paired sample *t* test). Additionally, the increase in interbrain correlation was not observed in experiments using the Thy1-GFP mouse line (Feng et al., 2000), which expresses a neuronal activity independent fluorophore in neurons, but is still susceptible to hemodynamic artifacts (*n* = 4 trials, *p* = 0.44, one-way ANOVA; Extended Data Fig. 3-1). An expanded view of the interaction period from Figure 3*b* is shown with behavior annotations in Figure 3*e*. Prominent calcium events are often accompanied by sustained periods of behavior (Fig. 3*e*). The duration of the behavior event, measured as the duration of each threshold-crossing movement detected in either forelimb or whisker ROIs (Extended Data Fig. 2-1), was positively correlated with the average ΔF/F_0_ during the behavior (*n* = 2075 behavior events, Spearman correlation *r* = 0.40, *p* = 6.0 × 10^−81^; Fig. 3*f*). Interbrain correlations on a region-by-region basis, including putative motor areas [whisker motor cortex (wM1), secondary motor cortex (M2), anterior lateral motor cortex (ALM)], sensory areas [primary visual cortex (V1), forelimb (FL), hindlimb (HL), and anterior barrel cortex (aBC), posterior barrel cortex (pBC), retrosplenial (RS)], and parietal association area (PTA; Fig. 3*g*), were greater during the interaction period compared with the separated periods, with no significant differences found between cortical regions (0.17 ± 0.03 together vs −0.013 ± 0.009 before and −0.019 ± 0.02 after interaction; two-way ANOVA with *post hoc* Bonferroni correction, *n* = 35 trials, trial phase: *F*_(1,680)_ = 136, *p* = 1.0 × 10^−28^; brain regions: *F*_(9,680)_ = 0.81, *p* = 0.61; interaction: *F*_(9,680)_ = 1.2, *p* = 0.30; Fig. 3*h*,*i*). Example seed pixel correlation maps are shown in Extended Data Figure 3-2. Intrabrain correlations also showed a slight but significant increase during the interaction period, with no significant effects observed across individual brain regions (two-way ANOVA with *post hoc* Bonferroni correction, *n* = 35 trials, trial phase: *F*_(1,680)_ = 44.1, *p* = 6.3 × 10^−11^; brain regions: *F*_(9,680)_ = 0.30, *p* = 0.98, interaction: *F*_(9,680)_ = 0.01, *p* = 1.0; Extended Data Fig. 3-3). Coherence between brains was calculated to determine the relationship between global cortical signals as a function of frequency. Time-varying coherence between brains revealed an increase in global signal coherence during the interaction phase at frequencies below 0.2 Hz (Fig. 3*j*). Experiments with physical barriers in place (opaque cardboard sheet or a transparent copper mesh) to prevent whisker-whisker contact between mice did not result in significant increases in region by region interbrain correlation during the interaction phase (opaque trials: 0.023 ± 1.3 together vs −0.024 ± 0.09 before and 0.03 ± 0.13 after, repeated measures ANOVA, *n* = 15, *F*_(2,28)_ = 1.0, *p* = 0.34; mesh trials: 0.09 ± 0.22 together vs −0.011 ± 0.11 before and −0.012 ± 0.11 after, repeated measures ANOVA *n* = 16, *F*_(2,30)_ = 0.022, *p* = 0.88; Fig. 3*k*,*l*). Although barriers reduced average intermouse correlation, it is possible that some animals can sense each other at a more considerable distance. However, no ultrasonic vocalizations were detected during these experiments (Extended Data Fig. 3-4). Videos of all intermouse interactions are available so that readers can also assess interactions as the mice approach each other (see Open Science Framework link https://osf.io/teshq/).

### Cortical representations of behavior events

The extent to which behavior and trial events (mice together, apart, etc.) co-vary with the stationary mouse’s cortical activity was assessed using ridge regression (Musall et al., 2019). A set of predictor variables, which included behaviors from the stationary mouse throughout the trial, behaviors from the moving mouse during the together period, and events associated with the trial structure (Fig. 4*a*) were used to model the cortical activity from the stationary mouse. The ridge regression analysis was performed on the pooled data from a specific mouse for all trials in the open interaction condition with no barrier (Fig. 4), as well as in the mesh barrier conditions that were expected to reduce whisker contact (Extended Data Fig. 4-1). Across nine open-interaction trials, the ridge regression (behavior to brain activity) model predicted on average 30% of the GCaMP signal variance across the dorsal cortex and reached up to 54% for some areas within somatosensory cortex (Fig. 4*b*). In the same mouse across four mesh barrier trials (Extended Data Fig. 4-3*a*), the model performance was similar: explaining on average 33% of the variance in the cortical GCaMP signal across the dorsal cortex and up to 53% in somatosensory cortex (Extended Data Fig. 4-1*b*). To determine each variable’s nonredundant contribution to the total explained variance, the explained variance was computed after randomly permuting each of the single model variables. The difference in explained variance (*ΔR*^2^) shows the unique contribution of the permuted variable toward explaining the variance in the data (Musall et al., 2019). Unique contributions for the stationary mouse’s forelimb movements showed strong linkages with somatosensory areas (Fig. 4*c*; Extended Data Figs. 4-1, 4-2, 4-3, 4-4). In the open interaction trials, whisking movements initiated by the stationary mouse differed depending on the context (alone vs together), with an increase in explained variance observed in the posterolateral areas of cortex near whisker sensory areas when the mice were together (Fig. 4*d*,*e*; Extended Data Figs. 4-1, 4-2). Behaviors of the moving mouse also had some contribution toward the cortical activity of the stationary mouse in the open interaction trials, where moving-mouse forelimb or whisker movements co-vary with posterolateral cortical areas near the whisker somatosensory network in the stationary mouse (Fig. 4*f*,*g*; Extended Data Figs. 4-1, 4-2). In contrast, in the mesh barrier trials, whisking together compared with separately did not exhibit a localized increase in explained variance in the posterolateral cortical areas (Extended Data Figs. 4-3, 4-4*d*,*e*). Additionally, partner behaviors explained little variance in the stationary mouse cortical activity when the barrier was present (Extended Data Figs. 4-3, 4-4*f*,*g*). In both open and mesh barrier trials, trial-associated events (for example stage translation and moving mouse approach/leave) had minimal contributions to the explained variance of the stationary mouse’s cortical GCaMP activity (Fig. 4*f–j*; Extended Data Figs. 4-1, 4-2, 4-3) and present as broadly distributed noise rather than discrete cortical maps containing known circuit motifs.

## Discussion

We introduce a flexible, low-cost Raspberry Pi-based mesoscale imaging platform (∼2750 USD per rig). The dual mouse imaging system can create reproducible interactions between mice that constrains some of the possible behaviors and timing because of the head-restrained and rail-based system. This type of head-restrained social interaction may be useful for studying the neural correlates of whisker investigation of social partners in barrel cortex. Our results indicate widespread correlated cortical activity between the brains of interacting mice. This synchrony is not associated with the mechanics or timing of the imaging paradigm as it was not present when trial-shuffled mouse pairs were examined; and is not attributed to hemodynamic artifacts in the fluorescence signal, as synchronization was not observed across pairs of GFP mice. The low frequency coherence observed between brains and the greater temporal overlap of behaviors when the mice are together, suggest that the interanimal cortical synchronization observed in this work may be driven by temporally correlated bouts of behavior (e.g., whisking or forelimb movements). Although previous work found that the magnitude of interbrain synchronization may convey information regarding the social status of one of the individuals (Jiang et al., 2015; Kingsbury et al., 2019), we did not find a relationship between social rank differences (determined by the tube test assay) and cortex-wide interbrain synchronization in our experiments. This discrepancy might be attributed to the head-restrained nature of the interaction; however, a more rigorous examination (with greater statistical power) of the social hierarchy may be needed to determine whether there is a relationship between social rank and interbrain correlation.

Using ridge regression (Musall et al., 2019), we assessed the degree to which different behavioral variables correlate with cortical GCaMP activity in a region-specific manner. The robust explained variance maps for forelimb and whisker movements in the stationary mouse are consistent with recent findings that movements drive widespread neural activity across the cortex. Although lower in magnitude, moving-mouse behaviors also contributed to cortical GCaMP activity variance in the stationary animal, and the regional distribution qualitatively resembled the explained variance maps observed when the stationary mouse whisks in the presence of a partner, but not alone, suggesting the engagement of different cortical regions during this context. This may reflect cortical activity arising from physical contact made between whiskers of the mice, as whisking against the mesh barrier similarly explained variance in GCaMP activity in the posterolateral regions of cortex.

One limitation of the presented work is that the frame rate of the behavior camera was not fast enough to clearly resolve whisker movements. Detailed analyses of whisker movements in mice typically use camera acquisition rates of ∼500 fps (Sofroniew et al., 2014; Mayrhofer et al., 2019). It is possible that some whisking events were missed by our analyses, or the precise timing of whisk initiation was not accurately resolved. However, false-negative error rates should presumably be consistent across experiments. Addition of a second behavior camera on the moving stage is also possible and would allow for characterization of the moving mouse’s behavior during periods when the mice are separated. Another important consideration for interpreting the results here is that mice must be head-restrained to be imaged and positioned properly. In a previous study, head-fixation was found to be aversive, but with training and habituation, stress recedes (Z.V. Guo et al., 2014), and rodents can even be trained to restrain themselves (Murphy et al., 2020; Scott et al., 2013; Aoki et al., 2017). Furthermore, recent work indicates that head-fixed animals can still exhibit high levels of task performance in water reaching tasks (Galiñanes et al., 2018), or other licking-based choice tasks (Murphy et al., 2020; International Brain Laboratory et al., 2021) when both head-fixed and nonhead-fixed task versions were directly compared.

We therefore present the results as an interaction that occurs in the context of head-fixation and caution that the observed brain dynamics may not reflect true naturalistic social touch behavior. Despite this, head-restraint facilitates consistent and reproducible interactions between animals, allowing for trial-averaging of behaviors. Recent development of a head-mounted mesoscopic camera allows for the exciting possibility to examine cortex-wide neural dynamics during more naturalistic social interactions in freely-moving mice (Rynes et al., 2021). Our intention in designing this system was to reduce visual stimuli by lighting the scene under infrared light. Under such conditions it is unclear how well the mice could visually perceive each other. Future experiments could investigate visual signals related to the expectation of an upcoming social interaction by incorporating appropriate ambient lighting to allow for clear visual detection of an approaching partner. Additional experiments can also incorporate simultaneous electrophysiological (Xiao et al., 2017) or fiber photometry (Ramandi et al., 2023) recordings in subcortical targets such as hypothalamus, ventral striatum, or hippocampus for example, to assess correlations between dorsal cortex and socially relevant activity in these deep structures (Dölen et al., 2013; Okuyama et al., 2016; Rogers-Carter et al., 2018; Walsh et al., 2018).

## Synthesis

Reviewing Editor: Nathalie Ginovart, University of Geneva Decisions are customarily a result of the Reviewing Editor and the peer reviewers coming together and discussing their recommendations until a consensus is reached. When revisions are invited, a fact-based synthesis statement explaining their decision and outlining what is needed to prepare a revision will be listed below. The following reviewer(s) agreed to reveal their identity: Ariel Gilad.

The authors have engaged in a very constructive revision of their manuscript, and both reviewers agreed that this revised version is significantly improved compared to the original submission. However, prior to final acceptance, authors must address a few minor comments raised by reviewers. Please find below the reviewer’s comments.

Reviewer 1:

In the revised version, the authors have addressed many of my concerns. However, some were addressed with additional explanations rather than presenting new data or analyses, given that the experiments could not be reconducted. Nevertheless, I believe this version effectively highlights the advantages of their developed approach. I still have several comments and recommendations for the final manuscript:

1) Could you provide a comprehensive description of the tube test procedure? It would be useful to know how many times each mouse pair is tested daily. Without specific data on the tube test, it’s challenging to evaluate the nature and quality of this test. For example, many labs might repeat the test ten times per mouse, incorporating a movable partition midway in the tube to avoid biases. A more detailed explanation would be beneficial.

2) I remain unconvinced that the Allen mouse atlas can be accurately aligned solely using the bregma. Relying on the bregma can be problematic since it’s not always reliable, and it presupposes that the orientation and size of all the mice brains match perfectly with the atlas’s orientation and size. A direct functional comparison might have been more illustrative. At the very least, the authors should overlay the registered atlas on their functional maps consistently. This overlay is notably absent in Figure 4 and Figure 3-2, yet it’s crucial to validate the depicted maps in relation to identified regions.

3) For Figure 4, it would be enlightening to include similar maps for the other mice, especially focusing on the interaction epochs, since only then can the second mouse be fully monitored.

4) On line 260, when comparing the distributions, please provide values for both distributions. Without these, the context for the stated p-value and test is unclear.

5) Ensure that citations and references are meticulously checked and corrected. There are evident inconsistencies and errors, like on lines 29-30 where “n.d.” appears or where only the initials of the first names are present in some references.

6) Also, please maintain consistency when citing panels, either using capital letters throughout or not.

Reviewer 2:

The authors have addressed all of my previous concerns. One small issue, could the authors explicitly mention that they use an RGB camera with different channels to correct for non-calcium dynamics. This was not mentioned in the text and I only found it in previous publications. Other than that, I fully support publication.

## Author Response

Reviewer 1:

In the revised version, the authors have addressed many of my concerns. However, some were addressed with additional explanations rather than presenting new data or analyses, given that the experiments could not be reconducted. Nevertheless, I believe this version effectively highlights the advantages of their developed approach. I still have several comments and recommendations for the final manuscript:

1) Could you provide a comprehensive description of the tube test procedure? It would be useful to know how many times each mouse pair is tested daily. Without specific data on the tube test, it’s challenging to evaluate the nature and quality of this test. For example, many labs might repeat the test ten times per mouse, incorporating a movable partition midway in the tube to avoid biases. A more detailed explanation would be beneficial.

Response: We performed the tube test on a subset of the animals used in this study (2 cages, 8 mice total) and we did include a removable partition midway in the tube to avoid biases. We repeated the tube test for 4 trials per cage. In one cage, the rank was stable with 3/4 trials yielding a consistent rank (Figure 1, cage 1). In the other cage, one mouse (Figure 2, cage 2, yellow line) had inconsistent rankings, but the other 3 mice formed a stable hierarchy with respect to each other (Figure 1, cage 2). We tested the relationship between the social rank and inter-brain correlations across mouse pairs by calculating the Pearson Correlation coefficient between Inter-brain PCCs and differences in the median social rank of each mouse (Figure 1, bottom).

We have included more details on the tube test methodology on pg 5 line 72: “Social rank was estimated using the tube-test assay (Fan et al. 2019) in a subset of 8 mice from 2 cages. Briefly, mice were introduced to either end of a narrow plexiglass tube (32cm long, 2.5cm inner diameter). Upon meeting in the middle, mice compete by pushing each other to get to the opposite side. The mouse which pushes the other back out of the tube is deemed the winner. A plastic barrier was inserted at the midpoint of the tube and was removed when both mice approached the middle to avoid biases. All combinations of mice within a cage were tested in a round robin format to determine the linear hierarchy. Tube test tournaments were repeated for four trials.”

We have also added to the discussion that a more detailed examination of the social hierarchy may be needed to determine whether there is a relationship between social rank and interbrain correlations. From pg 20 line 387: “This discrepancy might be attributed to the head-restrained nature of the interaction, however a more rigorous examination (with greater statistical power) of the social hierarchy may be needed to determine whether there is a relationship between social rank and interbain correlation.”

Because we did not find any significant relationship in this analysis, we did not include much details or explanation in our initial presentation of the manuscript (other than what we show above). However, we acknowledge that tube test trials have not been conducted enough times to observe a stable hierarchy as defined by Fan et al, 2019. If the reviewer suggests that the data/analysis are not sufficient, we will remove the relevant text from the manuscript which would not affect the core concept that we are presenting a system to image dorsal cortical activity from two mice during a constrained social interaction-like behavior.

2) I remain unconvinced that the Allen mouse atlas can be accurately aligned solely using the bregma. Relying on the bregma can be problematic since it’s not always reliable, and it presupposes that the orientation and size of all the mice brains match perfectly with the atlas’s orientation and size. A direct functional comparison might have been more illustrative. At the very least, the authors should overlay the registered atlas on their functional maps consistently. This overlay is notably absent in Figure 4 and Figure 3-2, yet it’s crucial to validate the depicted maps in relation to identified regions.

Response: We agree with the reviewer’s point that functional mapping of the cortex would be a better approach to align the brain to its corresponding anatomical areas, but unfortunately that data was not collected for this group of animals. However, the method we employed to align the Allen mouse atlas to the brain does not solely rely on the bregma, but also uses key points along the superior sagittal sinus and the space between the olfactory bulb and the frontal cortex. Although this method is not perfect, it does help to account for differences in orientation or size.

While we could not validate the atlas alignment method using functional mapping on these particular animals, we qualitatively assessed the accuracy of the method on other experiments (unrelated to this manuscript) where sensory stimulation was performed via hindlimb stimulation. See below figure: A) Evoked response map (dF/F) from stimulation of right hindlimb. B) Mean dF/F within the yellow box from A over time (stimulus presented on frame 300). C) Image of the cortical surface with the same region highlighted. D) Result of Allen atlas alignment using the methods employed in the manuscript. Blue arrows in C and D point to the same area across regions as determined by vessel branching patterns. The overlaid atlas showed overlap between the estimated hindlimb somatosensory cortex (D) and the measured hindlimb somatosensory cortex (A and C).

We have elaborated on this method and added some discussion of its limitations on page 11 line 193: “Individual regions were selected from coordinates with respect to bregma (Figure 3g), and their placement was verified after alignment with the Allen Institute Common Coordinate Framework brain atlas (Wang et al. 2020; Saxena et al. 2020), which utilized key-points along bregma, the superior sagittal sinus, and the space between the olfactory bulb and the frontal cortex. It should be noted that while these key-points can provide a reasonable alignment of the data with the cortical atlas, using key-points obtained with functional mapping may be a more accurate method.” We have also softened the wording throughout the manuscript in the presentation and discussion of results to be more conservative when discussing specific cortical regions, and overlaid the registered atlas over Figures 3-2, 4, 4-1, 4-2, 4-3, and 4-4.

In re-running the ROI based figures during the first round of revisions, there was a small change in the statistics for figures 3i and 3-3b resulting from a slight difference in ROI placement between the different rounds of analyses. However, these differences do not change any of the significance of the statistical analysis or reported findings. We have updated the original statistical values with the new values in the text and statistics table.

3) For Figure 4, it would be enlightening to include similar maps for the other mice, especially focusing on the interaction epochs, since only then can the second mouse be fully monitored.

Response: We have added extended data figures 4-1, 4-2, 4-3, and 4-4 which include examples of this analysis for other mice. The new figures further confirm the motor representations of forelimb movement events, and provide additional support that behaviors of the moving mouse in this head-restrained social interaction paradigm can evoke or correspond with dorsal cortical activation in the stationary mouse.

When running the analysis for the additional mice, we noticed an indexing error in our original code. While this error did not have a substantial effect on the key takeaways from the main figure, some of the findings from the initially submitted Extended Data figure (notably panels b and c) were inaccurate. We have corrected this mistake and updated the figures and corresponding text. These changes have not had a major effect on the narrative, and the additional examples with correct indexing provide further support for the findings summarized above.

We are thankful that the reviewer’s thorough evaluation brought this error to light, and its correction has ultimately improved the quality of our work.

4) On line 260, when comparing the distributions, please provide values for both distributions. Without these, the context for the stated p-value and test is unclear.

Response: We have provided values for both distributions.

5) Ensure that citations and references are meticulously checked and corrected. There are evident inconsistencies and errors, like on lines 29-30 where “n.d.” appears or where only the initials of the first names are present in some references.

Response: We have corrected all citations and references.

6) Also, please maintain consistency when citing panels, either using capital letters throughout or not.

Response: We have ensured that all figure panels are consistently referenced in the text with lower-case letters.

Reviewer 2:

The authors have addressed all of my previous concerns. One small issue, could the authors explicitly mention that they use an RGB camera with different channels to correct for non-calcium dynamics. This was not mentioned in the text and I only found it in previous publications. Other than that, I fully support publication.

Response: We have added further elaboration to the GCaMP Image Acquisition section of the Methods to clarify the use of an RGB camera.

From pg 7 line 116 “GCaMP activity was imaged using RGB Raspberry Pi Cameras... The GCaMP imaging cameras... were equipped with triple-bandpass filters (Chroma 69013m), which allowed for the separation of GCaMP epifluorescence signals and reflectance signals into the green and blue channels respectively.
