# Supplementary figures and images for: Meso-Py: Dual Brain Cortical Calcium Imaging in Mice during Head-Fixed Social Stimulus Presentation
Source: eNeuro. 2023 Dec 15;10(12):ENEURO.0096-23.2023. doi: 10.1523/ENEURO.0096-23.2023 (PMC10731520; doi:10.1523/ENEURO.0096-23.2023)

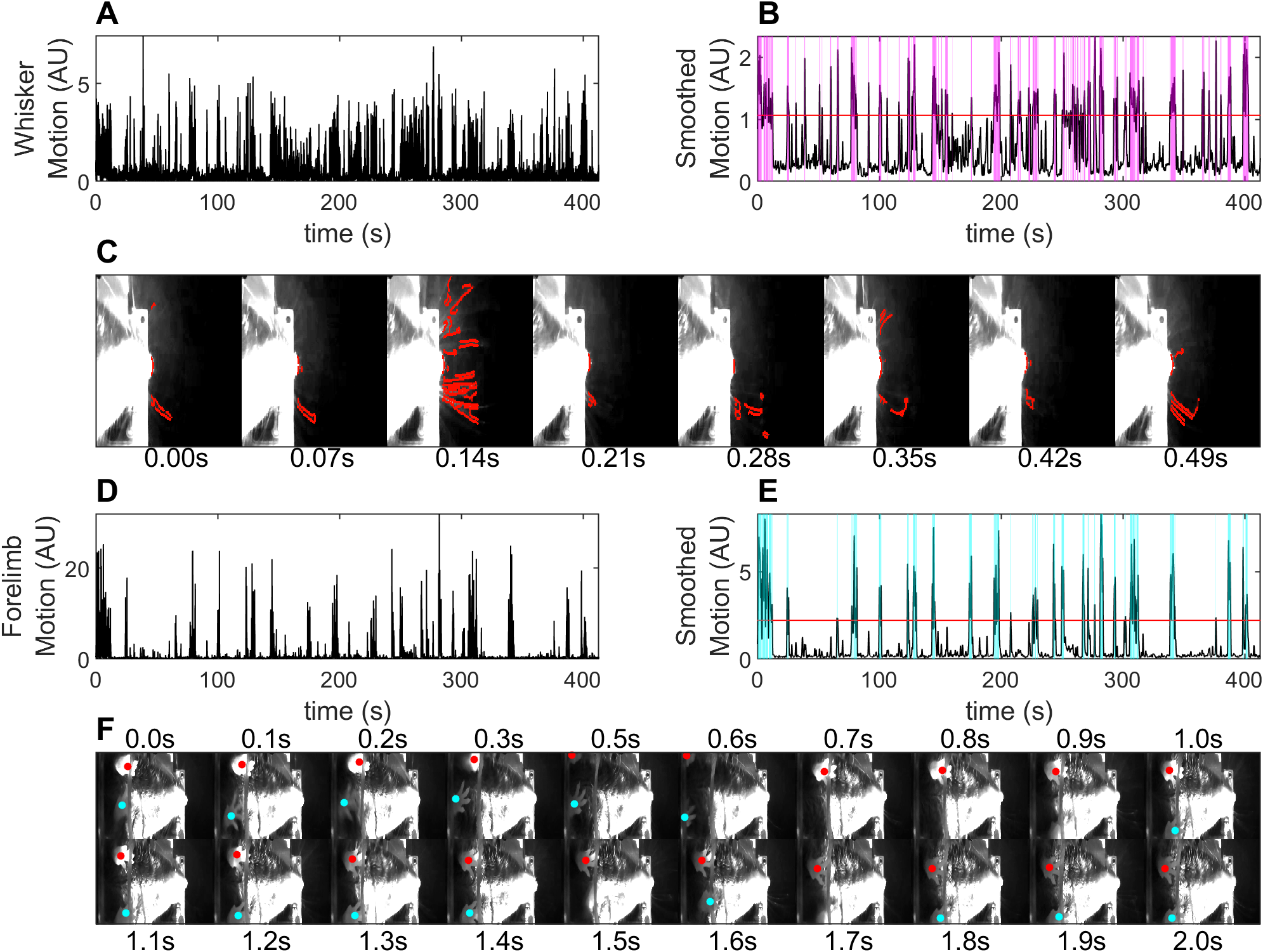

Supplement: Extended Data Figure 2-1 — Protocol for estimating behaviors. a, Raw motion energy within the whisker region of interest. b, Smoothed whisker motion energy. Motion energy exceeding a threshold of the mean + 1 SD (red line) is classified as binary movement behavior (shaded areas). c, Example montage of whisking behavior. Images are taken from beneath the mouse (see Figs. 1 and 2). Individual frames are cropped and displayed with saturated pixels, and a Canny edge detection algorithm was run over the whisker region to enhance visualization of whiskers. A whisker protraction event can be seen at 0.14 s. d–f, Same as a–c for forelimb movements. Individual frames are cropped to aid with visualization. Left and right paws are labelled with red and cyan markers, respectively. Download Figure 2-1, TIF file. [file enu-eN-MNT-0096-23-s01.tif]

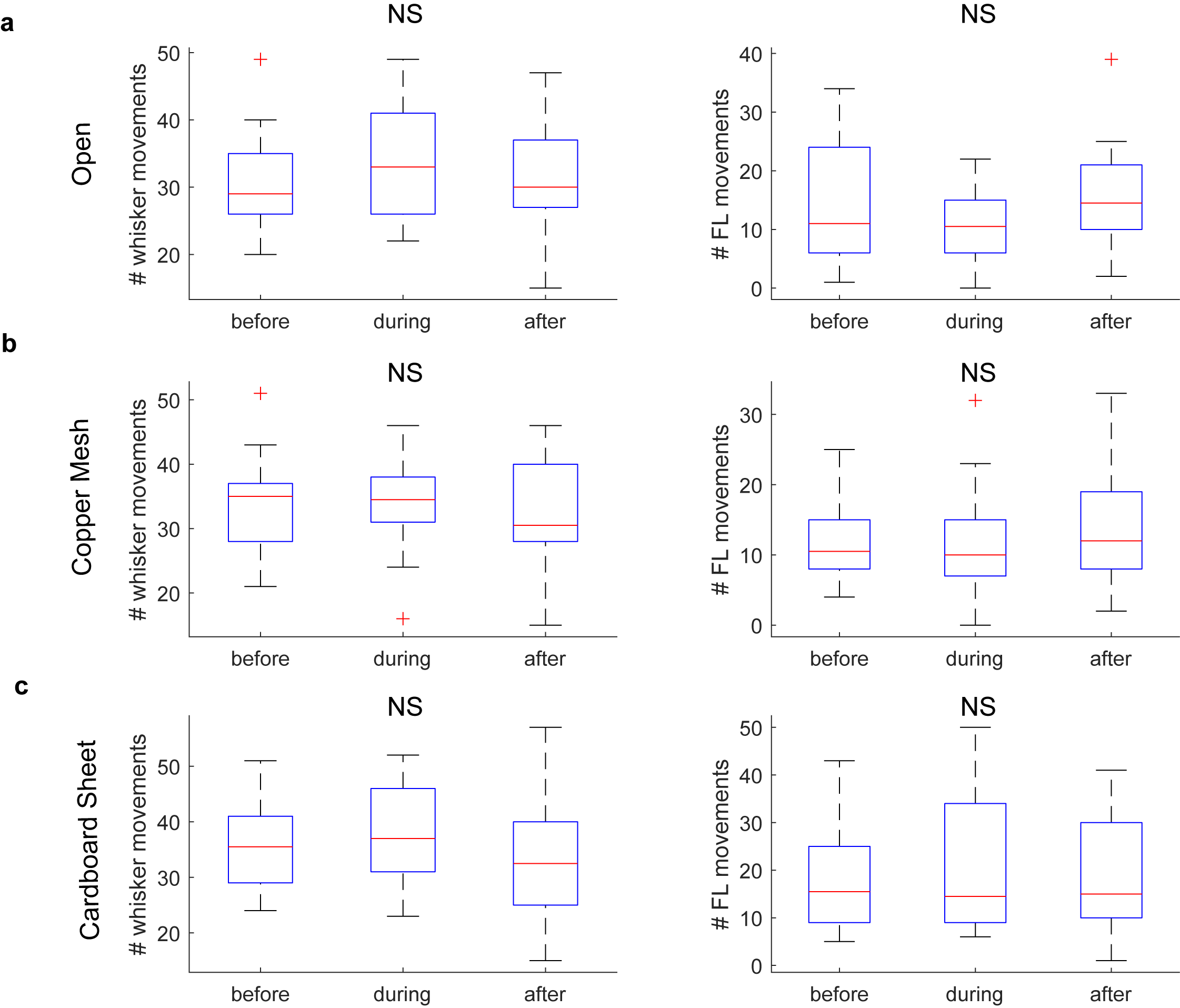

Supplement: Extended Data Figure 2-2 — Number of whisker or forelimb movements do not change between trial phases. Number of whisker movements (left) and forelimb movements (right) for all trial phases during open social interaction experiments (a) and barrier controls (b, c). During: during interaction period while mice are stationary and together (face to face); before/after: before/after interaction period while mice are stationary and apart. No significance between trial phases for all conditions (open n = 33 trials, mesh n = 16 trials, opaque n = 11 trials). Download Figure 2-2, TIF file. [file enu-eN-MNT-0096-23-s02.tif]

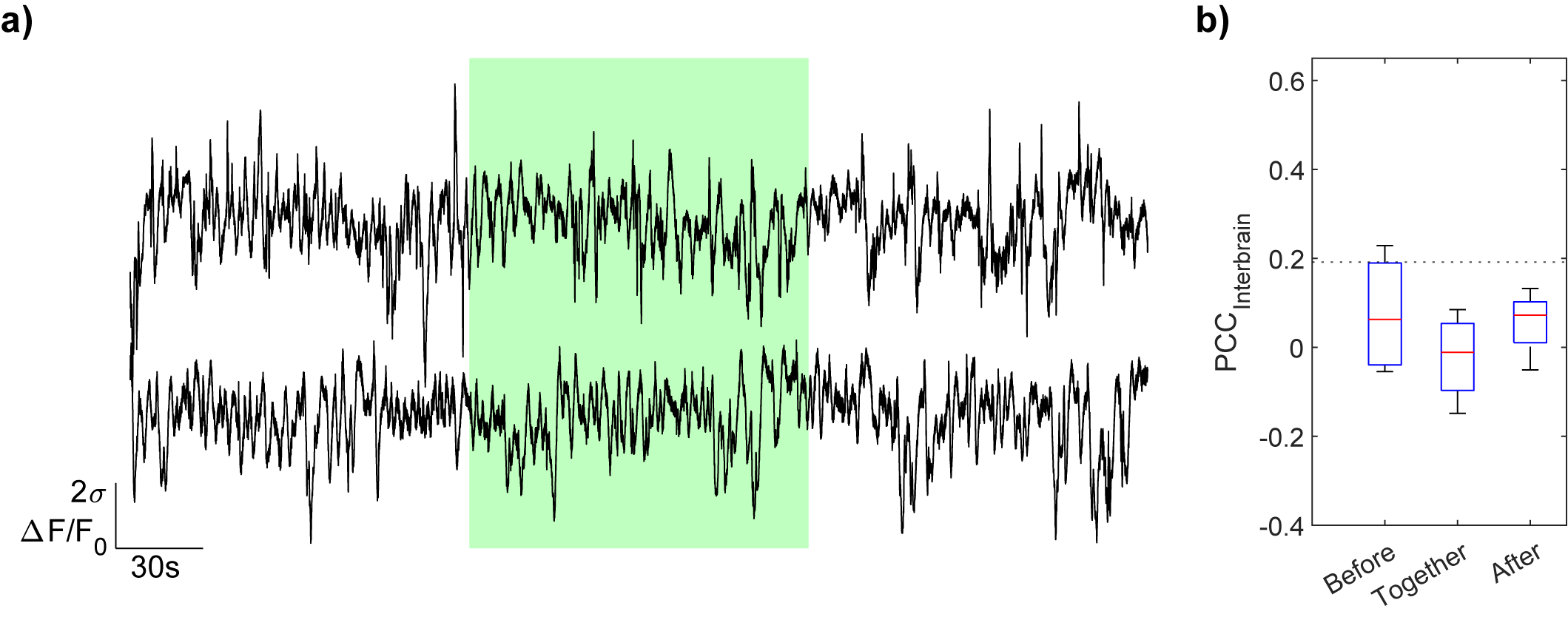

Supplement: Extended Data Figure 3-1 — No interanimal correlation observed in Thy1-GFP mice. a, Representative example of GFP activity global signals over the entire cortical mask for the stationary mouse (top) and moving mouse (bottom). Green shading indicates period when mice were together. b, Pearson correlation coefficients computed at each phase of the experiment. Dashed line shows median correlation coefficient between global signals for the GCaMP mice during the interaction phase of the experiment from Figure 3c. No significant difference was observed between phases. n = 4 trials, p = 0.4, one-way ANOVA. Download Figure 3-1, TIF file. [file enu-eN-MNT-0096-23-s03.tif]

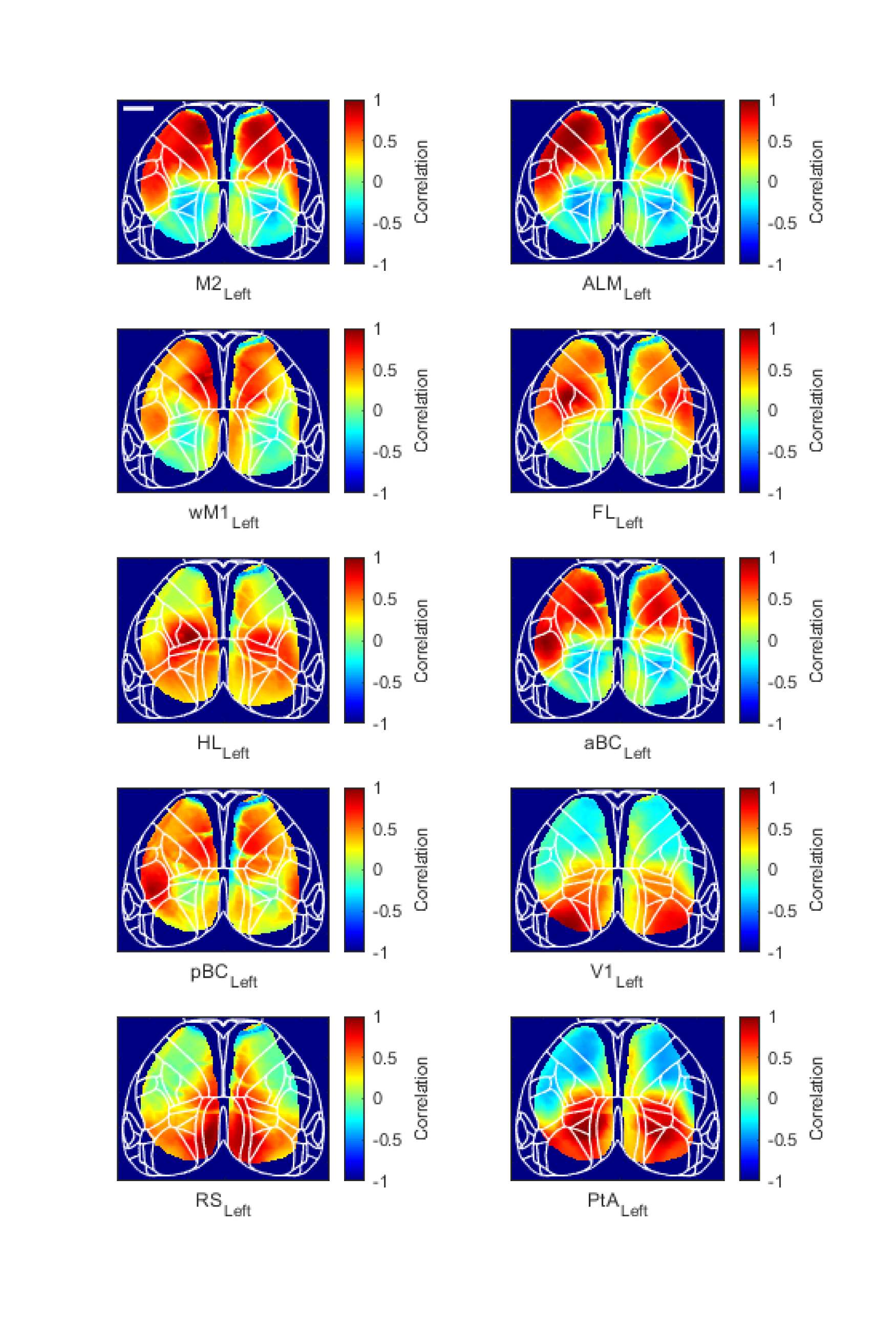

Supplement: Extended Data Figure 3-2 — Seed pixel correlation maps for putative cortical ROIs from an example mouse. Pearson correlation coefficients over the entire trial (spanning both separate and together phases) were calculated between every pixel within the cortical mask and the averaged signal obtained from a five by five-pixel neighborhood chosen from the specified region in each panel. Scale bar: 2 mm. Download Figure 3-2, TIF file. [file enu-eN-MNT-0096-23-s04.tif]

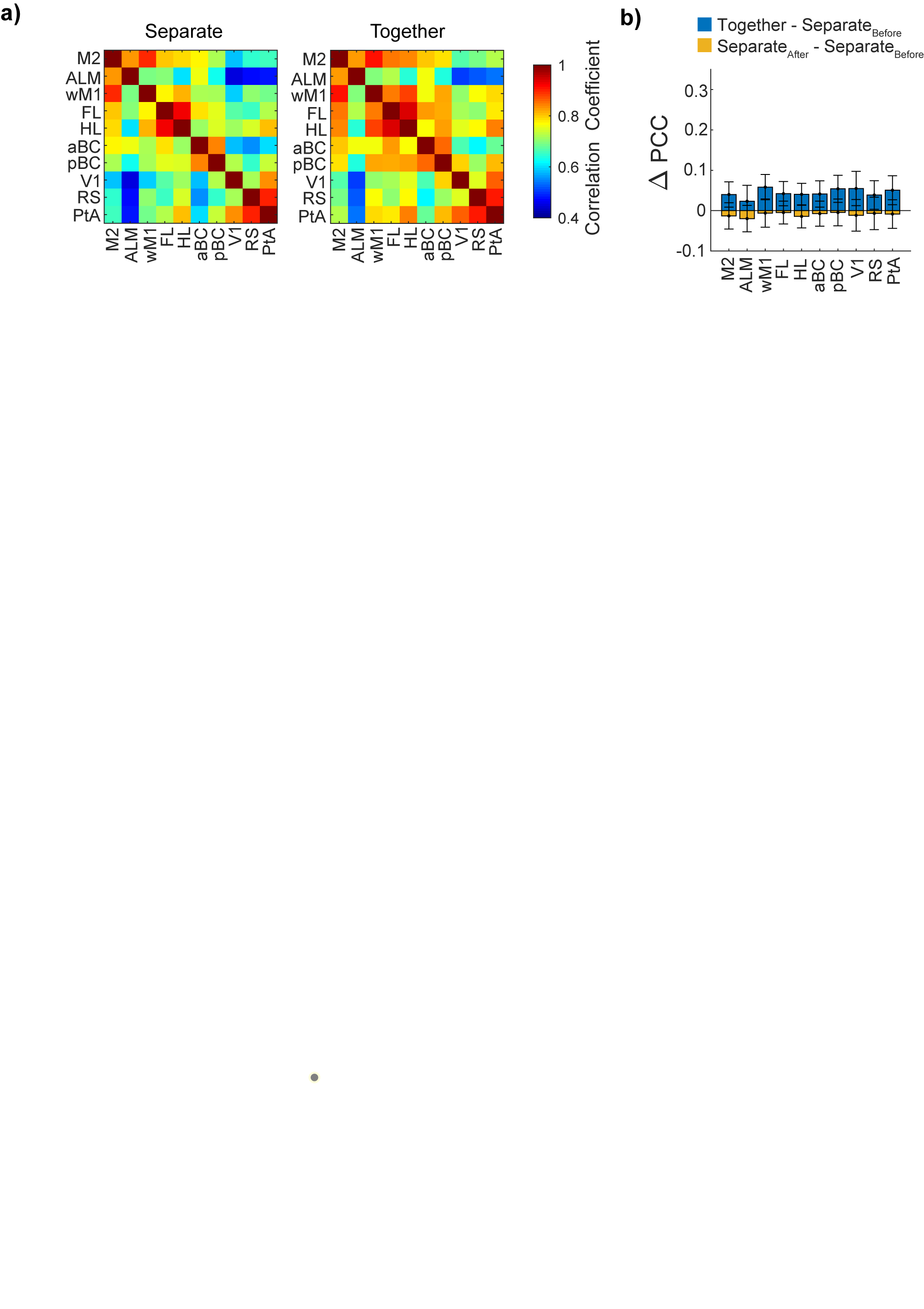

Supplement: Extended Data Figure 3-3 — Intrabrain correlations increase during interaction phase of the trial. a, Averaged intrabrain correlation matrices across all experiments during the period before interaction (left) and the period during interaction (right). b, Change in intrabrain correlation for each region of interest against all other regions, averaged across mice (n = 35 mouse pairs, *p < 0.05; two-way ANOVA with post hoc Tukey–Kramer test). Bars show mean ± SE; y-axis is scaled similarly to interbrain correlation changes from Figure 3i. Download Figure 3-3, TIF file. [file enu-eN-MNT-0096-23-s05.tif]

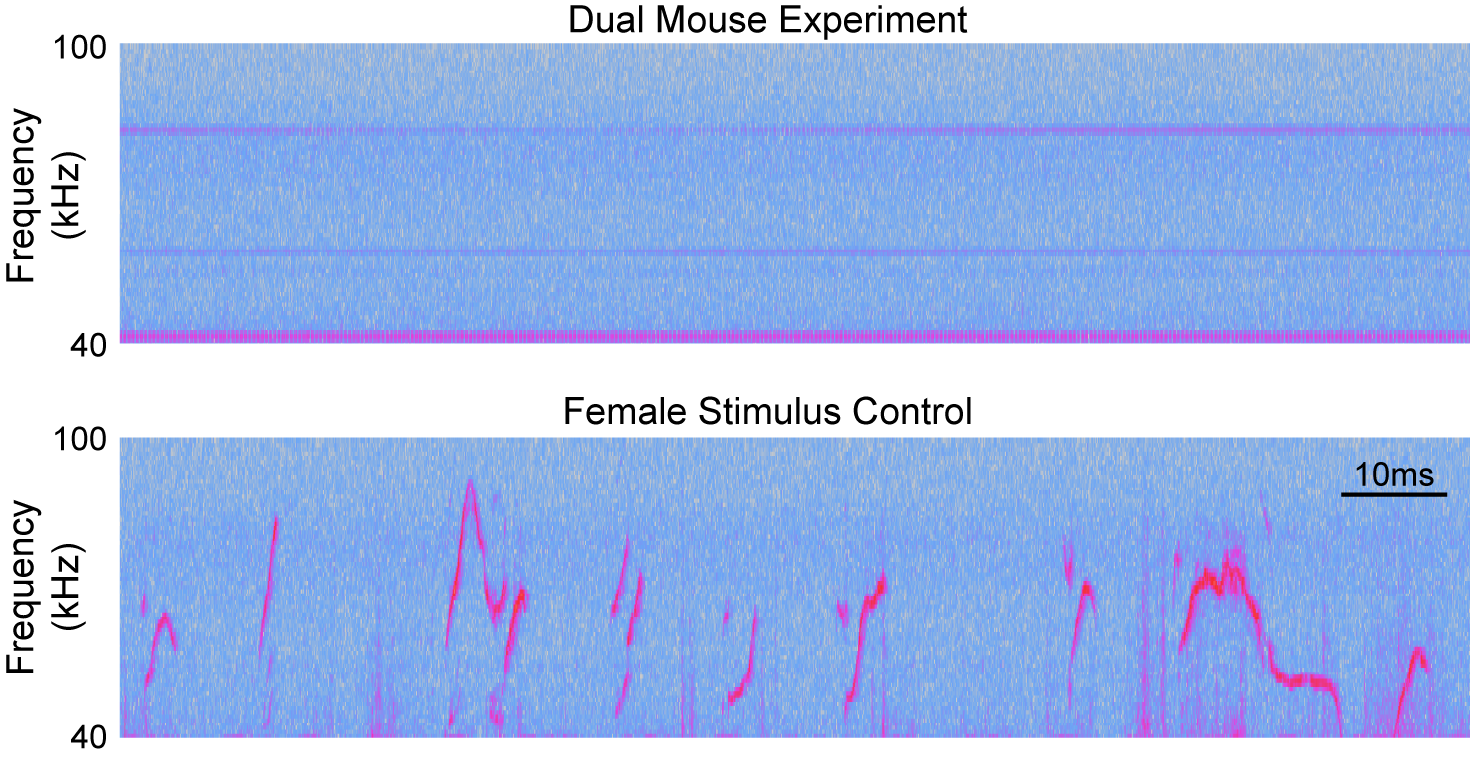

Supplement: Extended Data Figure 3-4 — No ultrasonic vocalizations detected during social-interaction tests. Example data from the social interaction experiment (top), compared to a control experiment taken from a breeder mouse introduced to a female (bottom). Ultrasonic vocalizations are clearly observed in the female stimulus control experiment, but not in the two-mouse imaging experiments. Download Figure 3-4, TIF file. [file enu-eN-MNT-0096-23-s06.tif]

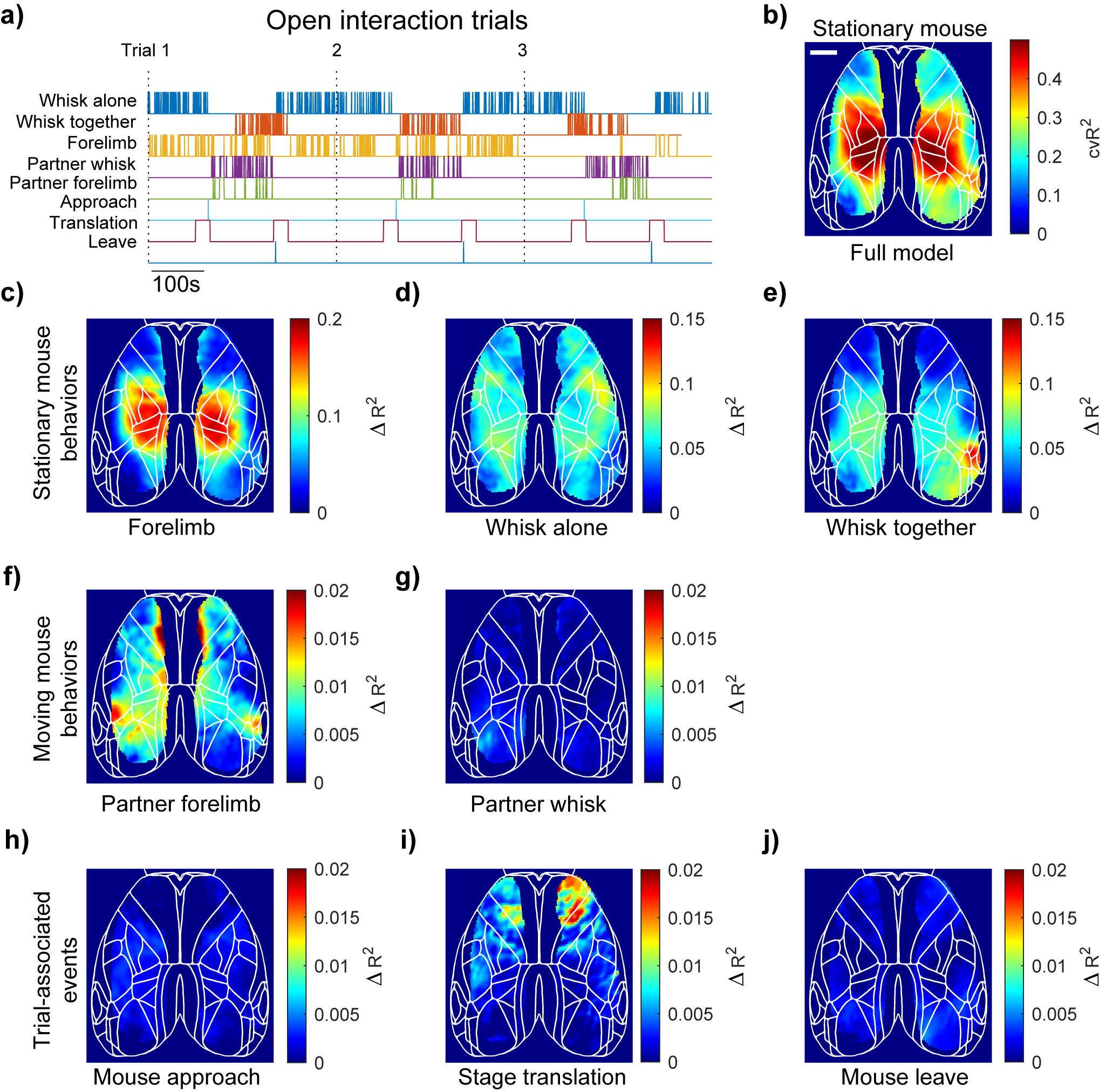

Supplement: Extended Data Figure 4-1 — Ridge regression model on an additional mouse in open interaction trials. a–j, Binary event vectors (colored lines) considered in the ridge regression model. Model included three separate interaction trials which had been concatenated together (dotted lines; partner limb and whisker behavioral variables were only assessed during the together phase). b, Explained variance for the full model after 10-fold cross-validation, projected back onto the cortical map. Scale bar: 2 mm. c–e, Unique contribution for each stationary mouse behavioral model variable; taken as the difference in explained variance between the full model and the reduced model with the specified variable randomly permuted. f, g, Same as c–e, except for the partner mouse behaviors. h–j, Same as c–e, except for trial-associated events. Download Figure 4-1, TIF file. [file enu-eN-MNT-0096-23-s07.tif]

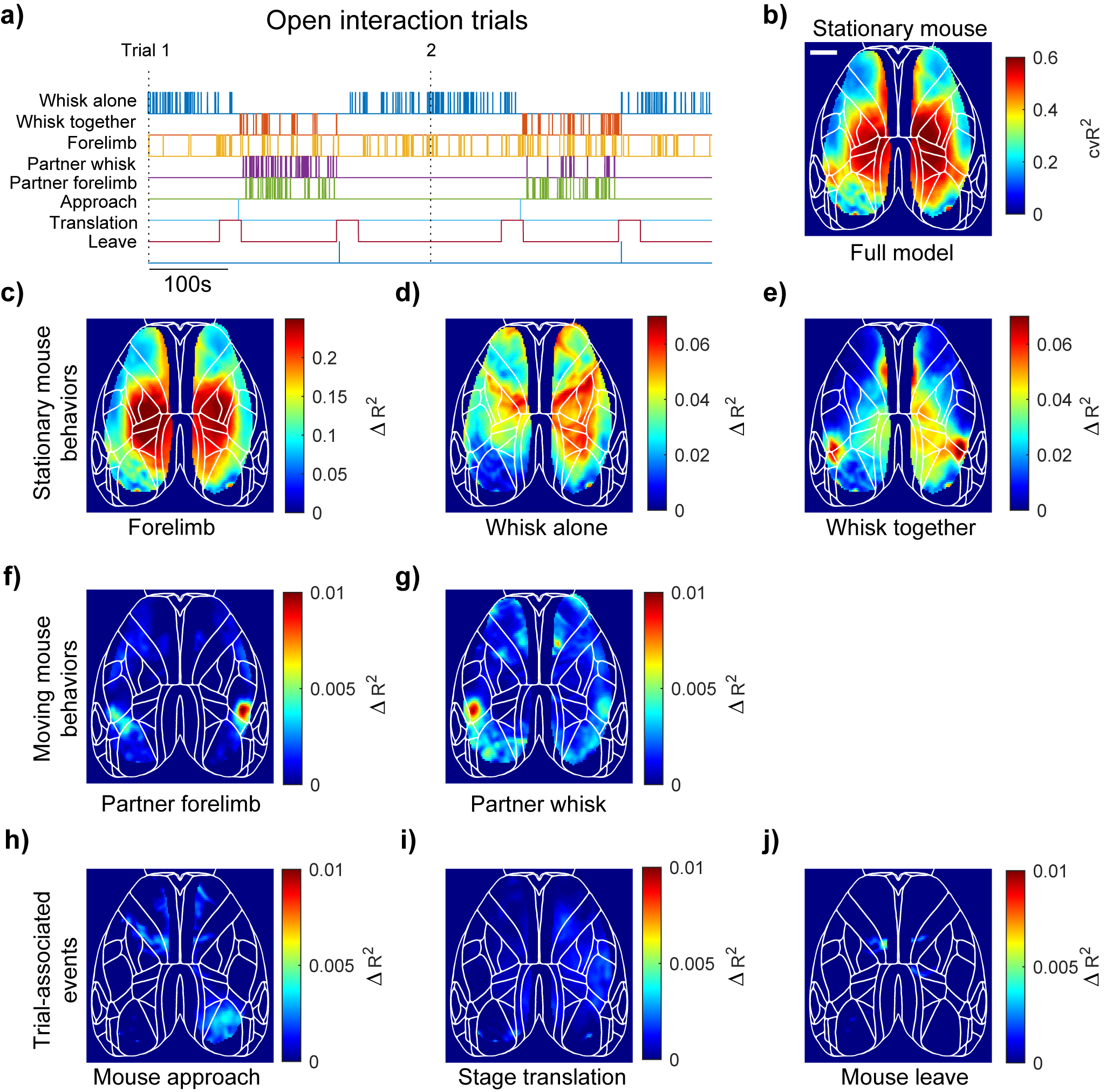

Supplement: Extended Data Figure 4-2 — Ridge regression model on an additional mouse in open interaction trials. a–j, Binary event vectors (colored lines) considered in the ridge regression model. Model included two separate interaction trials which had been concatenated together (dotted lines; partner limb and whisker behavioral variables were only assessed during the together phase). b, Explained variance for the full model after 10-fold cross-validation, projected back onto the cortical map. Scale bar: 2 mm. c–e, Unique contribution for each stationary mouse behavioral model variable; taken as the difference in explained variance between the full model and the reduced model with the specified variable randomly permuted. f, g, Same as c–e, except for the partner mouse behaviors. h–j, Same as c–e, except for trial-associated events. Download Figure 4-2, TIF file. [file enu-eN-MNT-0096-23-s08.tif]

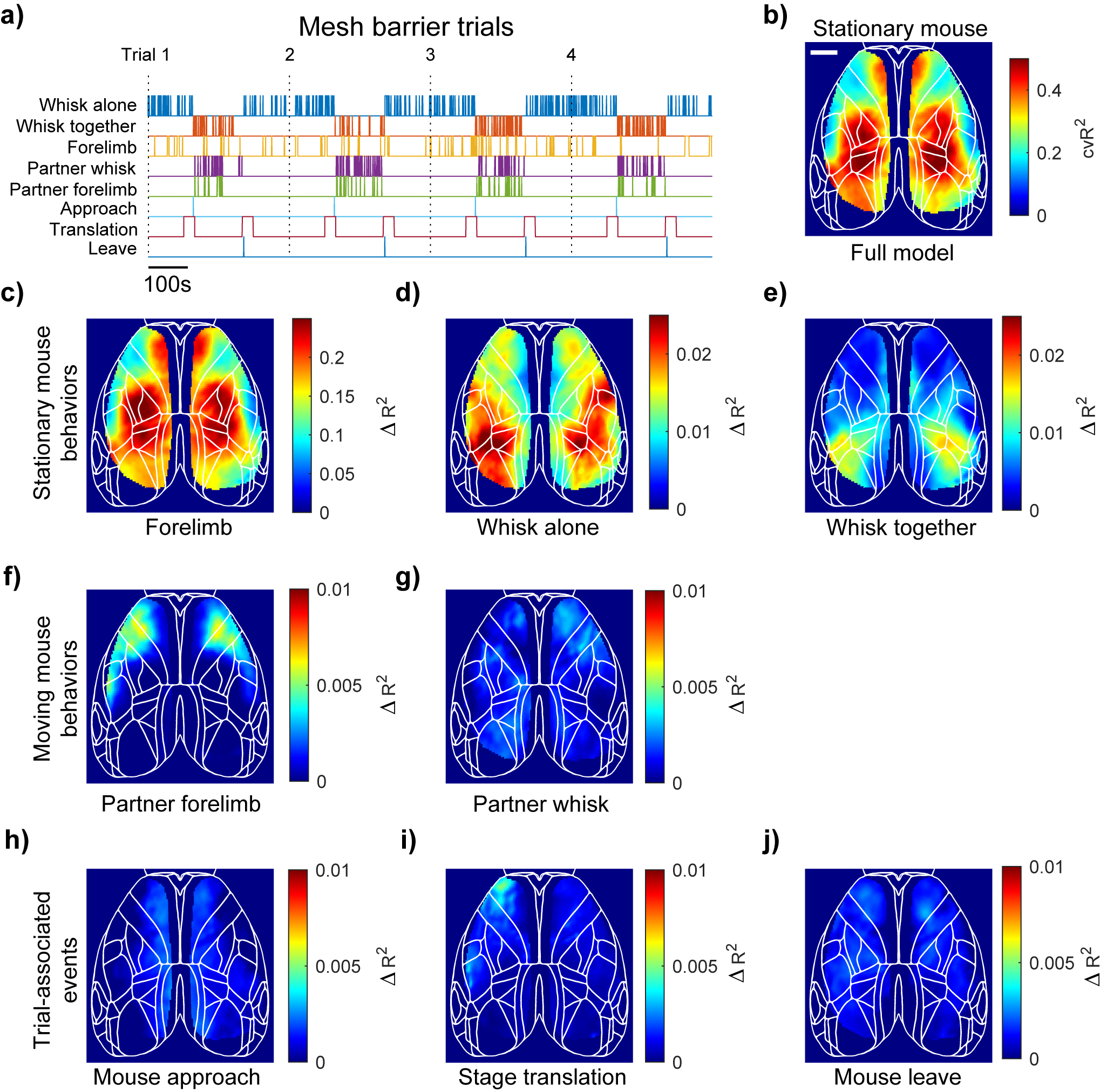

Supplement: Extended Data Figure 4-3 — Ridge regression model in mesh barrier trials. a, Binary event vectors (colored lines) considered in the ridge regression model. Model included four separate interaction trials which had been concatenated together (dotted lines; partner limb and whisker behavioral variables were only assessed during the together phase). b, Explained variance for the full model after 10-fold cross-validation, projected back onto the cortical map. Scale bar: 2 mm. c–e, Unique contribution for each stationary mouse behavioral model variable; taken as the difference in explained variance between the full model and the reduced model with the specified variable randomly permuted. f, g, Same as c–e, except for the partner mouse behaviors. h–j, Same as c–e, except for trial-associated events. Download Figure 4-3, TIF file. [file enu-eN-MNT-0096-23-s09.tif]

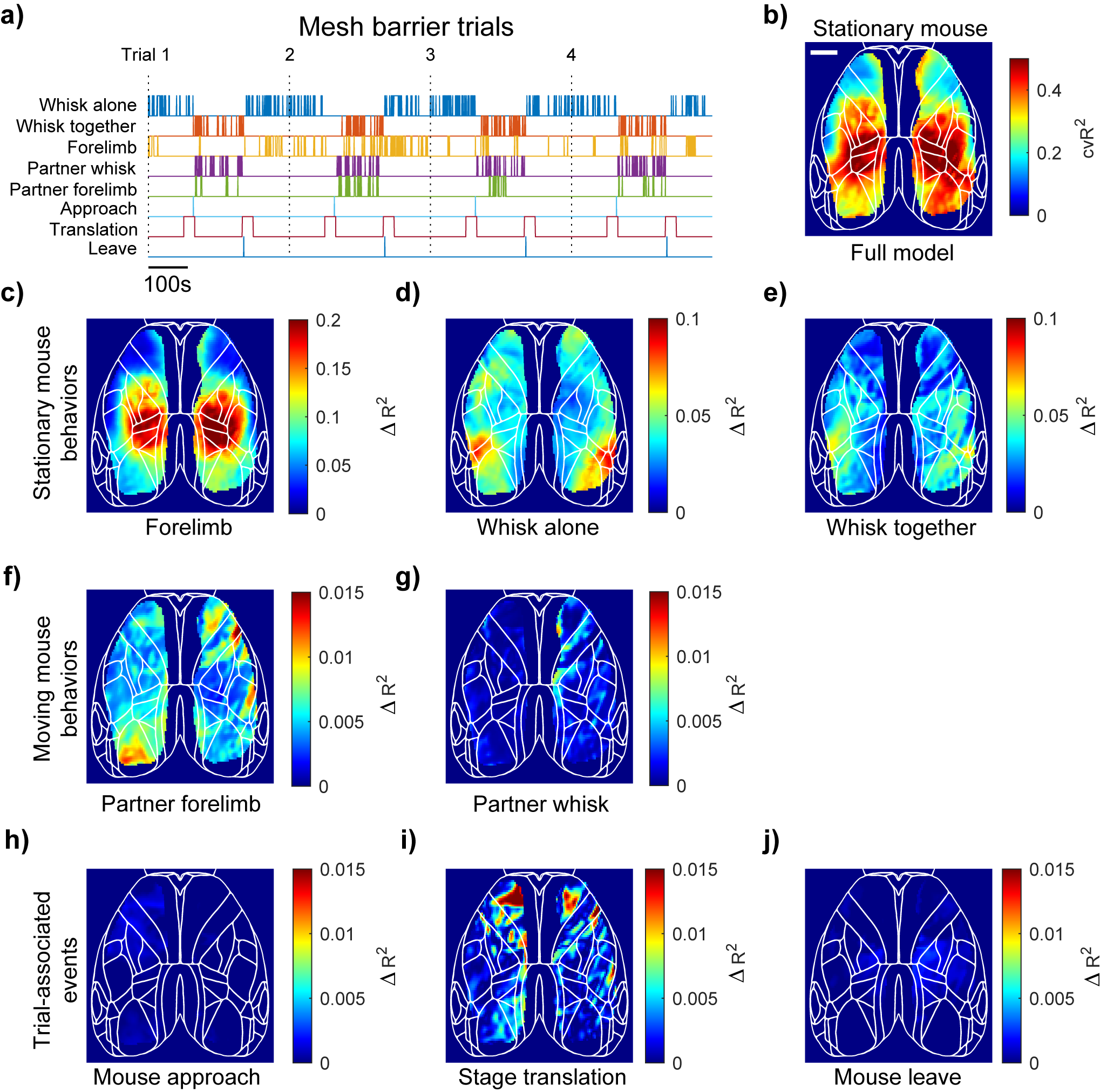

Supplement: Extended Data Figure 4-4 — Ridge regression model on an additional mouse in mesh barrier trials. a–j, Binary event vectors (colored lines) considered in the ridge regression model. Model included four separate interaction trials which had been concatenated together (dotted lines; partner limb and whisker behavioral variables were only assessed during the together phase). b, Explained variance for the full model after 10-fold cross-validation, projected back onto the cortical map. Scale bar: 2 mm. c–e, Unique contribution for each stationary mouse behavioral model variable; taken as the difference in explained variance between the full model and the reduced model with the specified variable randomly permuted. f, g, Same as c–e, except for the partner mouse behaviors. h–j, Same as c–e, except for trial-associated events. Download Figure 4-4, TIF file. [file enu-eN-MNT-0096-23-s10.tif]
